# Supplementary material for: Candidate Genes and Pathways Associated with Gilles de la Tourette Syndrome—Where Are We?
Source: Genes (Basel). 2021 Aug 26;12(9):1321. doi: 10.3390/genes12091321 (PMC8468358; doi:10.3390/genes12091321)
Supplement: Supplementary file 1 [file genes-12-01321-s001.zip › genes-1240846-supplementary.pdf]

Supplementary Table S1 – Gilles de la Tourette Syndrome Candidate Genes and Regions

Supplementary Table S1A

Gene	Chromosome	Finding	Sample Size (Ancestry)	Analysis	Reference
SLITRK1 (Slit and Trk-like 1)	13q31.1	Chromosomal inversion near *SLITRK1*; varCDfs and two occurrences of var321 identified in GTS probands.	4 families (GTS probands +/- ADHD or OCD); 174 GTS probands; 253 controls; 3,600 control chromosomes (Mostly Caucasian)	Breakpoint mapping, Sequence analysis, association	[1]
		Association between GTS and rs9593835 plus haplotypes of three tagging SNPs.	154 GTS nuclear families (Canadian)	Sequence analysis, association	[2]
			222 GTS trios (European)	Association (TDT)	[3]
			92 GTS (Japanese); 361 controls (NA)	Sequence analysis, association	[4]
		Support for Abelson et. al (2005).	1,117 GTS (European); 758 GTS (Jewish); 7 var321 carriers and their relatives	Genotyping, MDS, haplotype mapping	[5]
		Finding of known variants rs150504822 and rs146746846 plus novel variants c.1158C>A and c.1061C>G.	382 GTS (European, Canadian)	Sequence analysis	[6]
		Finding of three rare missense variants in *SLITRK1*.	120 GTS; 788 controls (French)	Sequence analysis	[7]
		Only c.3225T>C suggested as a rare variant, no other variants identified.	160 GTS children (Taiwanese)	Sequence analysis	[8]
		No association found between GTS and *SLITRK1*.	92 GTS; 192 controls (Austrian)	Sequence analysis, association	[9]
			1 large family (Italian)	Sequence-, haplotype-, CNV analysis	[10]
		No association found between GTS and var321 (or varCDfs [4]).	82 GTS (Caucasian)	Sequence analysis, association	[11]
			1,048 GTS/CTD + 1,252 relatives (Mostly Caucasian)	Association	[12]
			114 GTS probands + 193 parents (Costa Rican); 193 GTS probands + 322 parents	Association	[13]

Gene	Chromosome	Finding	Sample Size (Ancestry)	Analysis	Reference
			(Ashkenazi); 511 controls (Ashkenazi)		
			154 GTS nuclear families (Canadian)	Sequence analysis, association	[2]
			112 GTS +/- ADHD/OCD (Danish)	Sequence Analysis	[14]
			92 GTS (Japanese); 361 controls (NA)	Sequence analysis, association	[4]
DRD2 (Dopamine receptor D2)	11q23.2	Association between GTS and rs6279, rs1079597, and rs4648318 plus one five-marker haplotype.	69 GTS nuclear families (South American)	Association (TDT)	[15]
		Elevated methylation level of *DRD2* in GTS patients.	51 GTS; 51 controls (NA)	Methylation assay	[16]
		Association between GTS and the C allele of the H313H polymorphism.	151 GTS children; 183 control children (Taiwanese)	Association	[17]
		Association between GTS and the A1 allele of the *Taq*I polymorphism.	147 GTS; 314 controls (Caucasian)	Association	[18]
			225 GTS/CTD probands + 60 GTS/CTD relatives + 132 non-GTS/CTD relatives; 67 controls (Caucasian)	Association	[19]
			151 GTS children; 183 control children (Taiwanese)	Association	[17]
			523 GTS; 87 GTS probands + 152 relatives; 564 controls (Hispanic and non-Hispanic Caucasian, Asian)	Meta-analysis	[20]
		No association found between GTS and the *Taq*I polymorphism.	61 GTS trios (German)	Association (HRR)	[21]
			110 GTS trios (French Canadian)	Association (TDT)	[22]
		No association found between GTS severity and the *Taq*I polymorphism.	Four multigenerational GTS families (European)	Association	[23]
		No association found between GTS and *DRD2.*	Two multigenerational GTS families (European)	Linkage analysis	[24]
			166 indv. in 15 GTS families (NA)	Linkage analysis	[25]
			One 85 indv. pedigree with 29 GTS indv + 20 CMT indv (British)	Linkage analysis	[26]

Gene	Chromosome	Finding	Sample Size (Ancestry)	Analysis	Reference
			201 GTS (from families and singletons); 253 controls (USA); replication: 44 GTS (from families and singletons); 73 controls (Spain)	Genotyping, association (TDT)	[27]
DRD4 (Dopamine receptor D4)	11p15.5	Association between GTS and the 48 bp VNTR.	64 GTS/CMT trios (North America)	Linkage analysis (TDT)	[28]
			110 GTS trios (French Canadian)	Association (TDT)	[22]
			73 GTS; 218 GTS trios; 405 controls (NA)	Association (TDT, HRR), Meta-analysis	[29]
		Association between OCD with tics and the 48 bp VNTR.	61 OCD probands +/- tics; 47 controls (Mexican)	Association	[30]
		No association found between GTS and the 48 bp VNTR.	102 GTS trios (German)	Association (TDT)	[31]
			223 GTS; 737 controls (European)	Association	[32]
		No association found between GTS and the 48 bp VNTR, the 120 bp duplication polymorphism, nor any of the three SNPs rs74302, rs1800955, and rs936462.	103 GTS trios; 284 controls (Caucasian)	Association	[33]
		No association found between GTS and the 48 bp VNTR nor the 120 bp duplication polymorphism.	266 GTS +/- ADHD; 236 controls (Caucasian)	Association	[34]
		No association found between GTS and the 48 bp VNTR, a 12 bp indel, nor the $(G)_n$ mononucleotide repeat polymorphism.	5 GTS families (Canadian)	Linkage analysis	[35]
		No association found between GTS and *DRD4*.	One 85 indv. pedigree with 29 GTS indv + 20 CMT indv (British)	Linkage analysis	[26]
			201 GTS (from families and singletons); 253 controls (USA); replication: 44 GTS (from families and singletons); 73 controls (Spain)	Genotyping, association (TDT)	[27]
SLC6A3 (Sodium-dependent dopamine transporter)	5p15.32	Decreased methylation level of *SLC6A3* in GTS patients.	51 GTS; 51 controls (NA)	Methylation assay	[16]
		Association between GTS and the 10 allele of the 40 bp VNTR.	225 GTS/CTD probands + 60 GTS/CTD relatives + 132 non-	Association	[19]

Gene	Chromosome	Finding	Sample Size (Ancestry)	Analysis	Reference
			GTS/CTD relatives; 67 controls (Caucasian)		
			169 children of various disorders + siblings; 71 control children + siblings (Mostly Caucasian)	Linkage, Association (TDT)	[36]
		Association between tic severity and the 9 allele of the 40 bp VNTR.	103 GTS trios; 284 controls (Caucasian)	Association	[33]
		Association between GTS and the *Dde*1 polymorphism. No association found between GTS and the 40 bp VNTR, nor the three SNPs rs8179029, rs27048, and rs6348.	266 GTS +/- ADHD; 236 controls (Caucasian)	Association	[34]
		No association found between GTS and the 40 bp VNTR.	110 GTS trios (French Canadian)	Association (TDT)	[22]
		No association found between GTS and *SLC6A3.*	201 GTS (from families and singletons); 253 controls (USA); replication: 44 GTS (from families and singletons); 73 controls (Spain)	Genotyping, association (TDT)	[27]
*HDC (*Histidine decarboxylase)	15q21.2	The W317X mutation was identified in GTS individuals.	720 GTS (Caucasian, Hispanic, Asian); Two-generation GTS/OCD pedigree (Caucasian); 3,360 controls total (Mostly Caucasian)	Sequence analysis, linkage analysis	[37]
		Association between GTS and SNPs rs854150 and rs1894236.	520 nuclear GTS families (European, Canadian)	Association (TDT)	[38]
		Association between GTS and W317X/*HDC.*	9 W317X carriers w/tics from 1 family; 9 controls (NA)	Parallel analysis of patients and *HDC* KO mice	[39]
		Finding of novel rare variant c.1564A>C.	382 GTS (European, Canadian)	Sequence analysis	[6]
		Finding of rare variant c.1492A>G.	120 GTS; 788 controls (French)	Sequence analysis	[7]
		Identification of variants IVS1+52C>T, c.426C>A, and c.1743G>A in GTS patients, but no significant association of the three variants with GTS.	120 GTS; 240 controls (Han Chinese)	Sequence analysis, association	[40]

Gene	Chromosome	Finding	Sample Size (Ancestry)	Analysis	Reference
		No association found between GTS and SNPs rs854150, rs854151, and rs854157.	241 GTS trios (Han Chinese)	Association (TDT, HRR)	[41]
SLC6A4 (Solute carrier family 6 member 4)	17q11.2	Association between GTS and the 5-HTTLPR/rs25531 L_A allele and the 5-HTTLPR/rs25531/rs25532 L_{AC} haplotype. Variant I425V identified in TS+/-OCD family.	151 GTS+/-OCD probands; 858 controls (European American)	Association	[42]
		Association between GTS and *SLC6A4* mRNA expression in individuals with the 5-HTTLPR/rs25531/rs25532 L_{AC} haplotype.	72 GTS+/- OCD; 87 controls (Danish)	Genotyping, functional analysis	[43]
		SLC6A4 was identified as a high confidence risk gene.	13 GTS multiplex families (Caucasian)	WES, genotyping	[44]
		No association found between GTS and the 5-HTTLPR polymorphism.	52 GTS; 63 controls (Italian)	Association	[45]
			87 GTS; 311 controls (Caucasian)	Association	[46]
			108 GTS trios (Han Chinese)	Association (TDT, HRR)	[47]
		No potentially deleterious variants identified.	382 GTS (European, Canadian)	Sequence analysis	[6]
DRD3 (Dopamine receptor D3)	3q13.31	Association between GTS and the *Msc*I polymorphism.	139 GTS (NA); 91 controls (North American, European, non-Hispanic Caucasian)	Association	[48]
		No association found between GTS and rs6280.	160 GTS + parents (101 families); 90 controls (Han Chinese)	Association (TDT)	[49]
		No association found between GTS and the *Msc*I polymorphism.	110 GTS trios (French Canadian)	Association (TDT)	[22]
		No association found between GTS and the *Msc*I and *Bac*I polymorphisms.	16 nuclear GTS families (NA)	Family based association	[50]
		No association found between GTS and *DRD3*.	One 85 indv. pedigree with 29 GTS indv + 20 CMT indv (British)	Linkage analysis	[26]
			201 GTS (from families and singletons); 253 controls (USA); replication: 44 GTS (from families and singletons); 73 controls (Spain)	Genotyping, association (TDT)	[27]

Gene	Chromosome	Finding	Sample Size (Ancestry)	Analysis	Reference
AADAC (Arylacetamide deacetylase)	3q25.1	Association between GTS and *AADAC* deletions.	111 GTS; 73 controls (Caucasian)	Association, CNV analysis	[51]
			243 GTS (Danish); 1,571 controls (Danish); 1,181 GTS (European); 118,730 controls (European)	CNV analysis, Association, Meta-analysis	[52]
		Association between GTS and rs762169706.	200 GTS; 300 controls (Han Chinese)	Sequence analysis, Association	[53]
		Stop loss variant c.1198T>C identified in MZ DC GTS twins and father.	1 MZ twin family (1 GTS twin + GTS father) (American Indian/Caucasian)	WES, CNV analysis	[54]
		Over-transmission of the A allele of rs1042201 in GTS individuals.	162 GTS; 148 GTS trios; 128 controls (Southern + Eastern European)	Meta-analysis, TDT	[55]
		No association found between GTS and rs186388618.	200 GTS; 300 controls (Han Chinese)	Sequence analysis, Association	[53]
IMMP2L (Inner mitochondrial membrane peptidase, subunit 2)	7q31	*IMMP2L* found disrupted in breakpoint.	1 GTS male with [dup(7)(q22.1-q31.1)] (NA)	Breakpoint mapping	[56]
			1 GTS-like male with [t(2;7)(p24.2;q31)] and [del(7q31.1-7q31.2)] (Caucasian, British)	Breakpoint mapping	[57]
		Significantly more intragenic deletions found in GTS individuals than controls.	188 GTS; 316 controls (Danish)	CNV analysis	[58]
		Association between GTS and rs7795011.	162 GTS; 148 GTS trios; 128 controls (Southern + Eastern European)	Meta-analysis, TDT	[55]
		No coding mutations identified in neither GTS nor ASD individuals.	39 GTS; 95 multiplex ASD families (European)	Sequence analysis	[59]
		No association found between GTS, *IMMP2L* deletions, and mitochondrial function.	4 GTS indv. with deletion + 3 non-GTS parents with deletion; 3 GTS indv. without deletion; 4 non-GTS indv. without deletion (Danish)	Mitochondrial functional assays	[60]
DRD1 (Dopamine receptor D1)	5q35.2	No association between GTS and rs155417 nor the 5'UTR polymorphism.	148 GTS children; 83 controls (Taiwanese)	Association	[61]

Gene	Chromosome	Finding	Sample Size (Ancestry)	Analysis	Reference
		No association found between GTS and *DRD1*.	One multigenerational GTS family (Mennonite)	Linkage analysis	[62]
			One 85 indv. pedigree with 29 GTS indv + 20 CMT indv (British)	Linkage analysis	[26]
			50 GTS; 35 GTS+ADHD; 30 GTS+OCD; 50 controls (European)	Sequence analysis	[63]
			201 GTS (from families and singletons); 253 controls (USA); replication: 44 GTS (from families and singletons); 73 controls (Spain)	Genotyping, association (TDT)	[27]
BTBD9 (BTB domain containing 9)	6p21.2	Association between GTS and rs9357271, rs4714156, and rs9296249.	298 GTS trios; 24 GTS indv.; 290 controls (French Canadian)	Association	[64]
		Association between GTS and rs9296249.	110 GTS; 440 controls (Han Chinese)	Association	[65]
		No association found between GTS and rs4714156 and rs9357271.	110 GTS; 440 controls (Han Chinese)	Association	[65]
		No association found between GTS and rs9357271, rs4714156, and rs9296249.	162 GTS; 180 controls (Polish)	Association	[66]
		No potentially deleterious variants identified.	382 GTS (European, Canadian)	Sequence analysis	[6]
CELSR3 (Cadherin EGF LAG seven-pass G-type receptor 3)	3p21.31	Damaging variants identified in GTS individuals.	325 GTS trios; 625 control trios; replication: 186 GTS trios (Mixed)	WES	[67]
		CELSR3 was identified as a high confidence GTS risk gene.	802 GTS trios; 1,184 control quartets (Mixed)	WES	[68]
		CELSR3 variants identified in two GTS individuals.	120 GTS; 788 controls (French)	Sequence analysis	[7]
		A damaging variant identified in a GTS proband.	15 GTS trios (Chinese); 1,153 control trios (SSC)	WES	[69]
DBH (Dopamine beta-hydroxylase)	9q34.2	Association between GTS and the B1 allele of the *Taq* B polymorphism.	225 GTS/CTD probands + 60 GTS/CTD relatives + 132 non-GTS/CTD relatives; 67 controls (Caucasian)	Association	[19]

Gene	Chromosome	Finding	Sample Size (Ancestry)	Analysis	Reference
		No linkage found between GTS and *DBH*.	One 85 indv. pedigree with 29 GTS indv + 20 CMT indv (British)	Linkage analysis	[26]
		No association found between GTS and the *Taq*I polymorphism, the $(CA)_n$ polymorphism, or the 19 bp indel. Association between GTS and the 19 bp indel in the Turkish sample is a likely false positive.	29 nuclear GTS families (Turkish); 42 nuclear GTS families; 5 large multigenerational families with 40 GTS indv. (Canadian).	Linkage, Association	[70]
		No association found between GTS and the *Taq*I polymorphism.	266 GTS +/- ADHD; 236 controls (Caucasian)	Association	[34]
TPH2 (Tryptophan hydroxylase 2)	12q21.1	Association between GTS and the C allele of rs4565946.	98 GTS; 178 controls (German)	Association	[71]
		Association between TD and the TT genotype of rs4565946. Association between tic severity and the G allele of rs4570625.	149 TD children; 125 controls (Han Chinese)	Association	[72]
		Nominally significant association between GTS and the T allele of rs4565946.	465 GTS/CTD probands in 412 families (USA, Europe, South Korea); Replication: 1,285 GTS; Replication: 4,964 controls	Association (TDT)	[73]
		No association found between GTS and rs4570625.	98 GTS; 178 controls (German)	Association	[71]
NRXN1 (Neurexin 1)	2p16.3	Association between GTS and *NRXN1* deletions.	111 GTS; 73 controls (Caucasian)	Association, CNV analysis	[51]
			210 GTS; 285 controls; 53 GTS (Latin American)	Association, CNV analysis	[74]
			2,434 GTS; 4,093 controls (European)	Association, CNV analysis	[75]
FN1 (Fibronectin 1)	2q35	Damaging variants identified in GTS individuals.	325 GTS trios; 625 control trios; replication: 186 GTS trios (Mixed)	WES	[67]
		FN1 was identified as a probable GTS risk gene.	802 GTS trios; 1,184 control quartets (Mixed)	WES	[68]
		De novo missense mutation identified in *FN1* in GTS individual.	119 GTS+ASD children; 2,603 ASD children (SSC)	GSEA	[76]

Gene	Chromosome	Finding	Sample Size (Ancestry)	Analysis	Reference
DNAJC13 (DnaJ (Hsp40) homolog subfamily C member 13)	3q22.1	Identification of novel variant A2057S.	7 GTS/CTD indv. in a 3-generation pedigree (European); 94 GTS/CTD; 100 controls (NA)	WES	[77]
		DNAJC13 was identified as a high confidence risk gene.	13 GTS multiplex families (Caucasian)	WES, genotyping	[44]
		The A2057S variant was not identified in any GTS/CTD individuals.	132 GTS/CTD (Han Chinese)	Genotyping	[78]
GDNF (Glial cell line-derived neurotrophic factor)	5p13.2	Association between GTS and rs3096140.	201 GTS (from families and singletons); 253 controls (USA); replication: 44 GTS (from families and singletons); 73 controls (Spain)	Genotyping, association (TDT)	[27]
		No association found between GTS and SNPs rs7731209, rs2973050, rs2910797, rs1549250, rs2216711, rs2973041, rs12518844, and rs3812047.	1 large GTS family (Canadian); 241 GTS children in 171 nuclear families (NA)	Linkage, Association (TDT)	[79]
		No association found between GTS and *GDNF*.	162 GTS; 148 GTS trios; 128 controls (Southern + Eastern European)	Meta-analysis, TDT	[55]
HCN1 (Hyperpolarization activated cyclic nucleotide gated potassium channel 1)	5p12	Association between GTS and rs1501361 {g}.	232,964 GTS/AN/ADHD/ASD/BP/MD/OCD/SCZ (incl. 4,645 GTS (European, North American)); 494,162 controls (Mixed)	GWAS	[80]
		The statistical significance of the ligand-gated ion channel signaling gene set in GTS was driven partly by SNP rs9790873 in *HCN1*.	3,581 GTS (European incl. Ashkenazi Jewish and French Canadian (+Latin American)); 7,682 ancestry matched controls	Genome-wide pathway analysis	[81]
		No association between GTS and SNPs rs16902086, rs994092, rs11743392, rs2589162, and rs7722380.	1 large GTS family (Canadian); 241 GTS children in 171 nuclear families (NA)	Linkage, Association (TDT)	[79]
HTR1A (5-hydroxytryptamine receptor 1A)	5q12.3	No association found between GTS and the C1019G polymorphism.	87 GTS; 311 controls (Caucasian)	Association	[46]
			43 GTS; 25 controls (German)	Sequence analysis	[82]

Gene	Chromosome	Finding	Sample Size (Ancestry)	Analysis	Reference
		Two missense mutations identified in two GTS individuals.	56 GTS; 20 controls (Canadian)	Sequence analysis	[83]
CNR1 (Cannabinoid receptor 1)	6q15	Association between GTS and the C allele of rs2023239. No association found between GTS and the three SNPs rs2180619, rs806379, and rs1049354.	262 GTS; 279 controls (Polish)	Association	[84]
		No association found between GTS and *CNR1*.	201 GTS (from families and singletons); 253 controls (USA); replication: 44 GTS (from families and singletons); 73 controls (Spain)	Genotyping, association (TDT)	[27]
		No association found between GTS and the three variants c.1326T>A, c.1359G>A, and c.1419+1G>C.	Three cohorts: 40 GTS; 81 controls; 56 GTS; 55 controls; 64 GTS; 66 controls (NA)	Association	[85]
CNTNAP2 (Contactin associated protein 2)	7q35	*CNTNAP2* found disrupted in breakpoint.	1 family with two GTS+OCD children, both [der(7)ins(7;2)(q35-q36;p21p23)] and an OCD father [inv(2)(p23q22), ins(7;2)(q35-q36;p21p23)].	Breakpoint mapping	[86]
		The statistical significance of the cell adhesion and transsynaptic signaling gene set in GTS was driven partly by *CNTNAP2.*	3,581 GTS (European incl. Ashkenazi Jewish and French Canadian (+Latin American)); 7,682 ancestry matched controls	Genome-wide pathway analysis	[81]
		The translocation breakpoint disrupts *CNTNAP2* in a non-GTS family, indicating no association between GTS and *CNTNAP2*.	1 non-GTS multigenerational family with [t(7;15)(q35;q26.1)]	Breakpoint mapping	[87]
COMT (catechol-O-methyltransferase)	22q11.21	No association found between GTS and the Val158Met.	52 GTS; 63 controls (Italian)	Association	[45]
		No association found between GTS and *COMT* methylation levels.	51 GTS; 51 controls (NA)	Methylation assay	[16]
		No association found between GTS and *COMT*.	201 GTS (from families and singletons); 253 controls (USA); replication: 44 GTS (from	Genotyping, association (TDT)	[27]

Gene	Chromosome	Finding	Sample Size (Ancestry)	Analysis	Reference
			families and singletons); 73 controls (Spain)		
MAOA (Monoamine oxidase A)	Xp11.3	Association between GTS and the VNTR near exon 1.	319 GTS probands + relatives; 57 controls (Caucasian)	Association	[88]
			110 GTS trios (French Canadian)	Association (TDT)	[22]
		No association found between GTS and *MAOA*.	201 GTS (from families and singletons); 253 controls (USA); replication: 44 GTS (from families and singletons); 73 controls (Spain)	Genotyping, association (TDT)	[27]
ACP1 (Acid phosphatase 1)	2p25.3	Association between GTS with comorbidities and the ACP*1A polymorphism.	50 GTS; 134 GTS+ADHD; 539 controls (European Caucasian)	Association	[89]
		No association found between GTS and the ACP*1A polymorphism.	266 GTS +/- ADHD; 236 controls (Caucasian)	Association	[34]
PKP4 (Plakophilin 4)	2q24.1	The statistical significance of the cell adhesion and transsynaptic signaling gene set in GTS was driven partly by *PKP4.*	3,581 GTS (European incl. Ashkenazi Jewish and French Canadian (+Latin American)); 7,682 ancestry matched controls	Genome-wide pathway analysis	[81]
		PKP4 was associated with GTS, ADHD and ASD.	93,294 GTS/ADHD/ASD/OCD (Mixed); 51,311 controls (Mixed)	Cross-disorder GWAS meta-analysis	[90]
PNKD (PNKD metallo-beta-lactamase domain containing)	2q35	Deleterious variants identified in GTS/CTD individuals.	6 indv. in 3-gen multiplex GTS/CTD family (NA)	WES	[91]
		PNKD was identified as a high confidence risk gene.	13 GTS multiplex families (Caucasian)	WES, genotyping	[44]
CADM2 (Cell adhesion molecule 2)	3p12.1	The statistical significance of the cell adhesion and transsynaptic signaling gene set in GTS was driven partly by *CADM2.*	3,581 GTS (European incl. Ashkenazi Jewish and French Canadian (+Latin American)); 7,682 ancestry matched controls	Genome-wide pathway analysis	[81]
		CADM2 was associated with GTS and OCD.	93,294 GTS/ADHD/ASD/OCD (Mixed); 51,311 controls (Mixed)	Cross-disorder GWAS meta-analysis	[90]

Gene	Chromosome	Finding	Sample Size (Ancestry)	Analysis	Reference
MRPL3 (Mitochondrial ribosomal protein L3)	3q22.1	Identification of novel variant S75N.	7 GTS/CTD indv. in a 3-generation pedigree (European); 94 GTS/CTD; 100 controls (NA)	WES	[77]
		The S75N variant was not identified in any GTS/CTD individuals.	132 GTS/CTD (Han Chinese)	Genotyping	[78]
DRD5 (Dopamine receptor D5)	4p16.1	No association found between GTS and *DRD5.*	201 GTS (from families and singletons); 253 controls (USA); replication: 44 GTS (from families and singletons); 73 controls (Spain)	Genotyping, association (TDT)	[27]
		No linkage found between GTS and *DRD5*.	One 85 indv. pedigree with 29 GTS indv + 20 CMT indv. (British)	Linkage analysis	[26]
GABRG1 (Gamma-aminobutyric acid type A receptor subunit gamma1)	4p12	Missense variant identified in GTS individuals.	15 GTS trios (Chinese); 1,153 control trios (SSC)	WES	[69]
		The statistical significance of the ligand-gated ion channel signaling gene set in GTS was driven partly by SNP rs1391174 in *GABRG1.*	3,581 GTS (European incl. Ashkenazi Jewish and French Canadian (+Latin American)); 7,682 ancestry matched controls	Genome-wide pathway analysis	[81]
NIPBL (Nipped-B-like)	5p13.2	Damaging variants identified in GTS individuals.	325 GTS trios; 625 control trios; replication: 186 GTS trios (Mixed)	WES	[67]
		NIPBL was identified as a probable GTS risk gene.	802 GTS trios; 1,184 control quartets (Mixed)	WES	[68]
SLC1A3 (Solute carrier family 1 member 3)	5p13.2	Identification of the E219D variant, no statistically significant association with GTS.	256 GTS; 224 controls (Caucasian)	Sequence analysis, association	[92]
		No association found between GTS and SNPs rs3776581, rs2269272, and rs2032893.	1 large GTS family (Canadian); 241 GTS children in 171 nuclear families (NA)	Linkage, Association (TDT)	[79]
WWC1 (WW and C2 domain containing 1)	5q34	Damaging variants identified in GTS individuals.	325 GTS trios; 625 control trios; replication: 186 GTS trios (Mixed)	WES	[67]

Gene	Chromosome	Finding	Sample Size (Ancestry)	Analysis	Reference
		WWC1 was identified as a high confidence GTS risk gene.	802 GTS trios; 1,184 control quartets (Mixed)	WES	[68]
OFCC1 (Orofacial cleft 1 candidate 1)	6p24.2	Identification of novel variants R129G and c.-5A>G.	7 GTS/CTD indv. in a 3-generation pedigree (European); 94 GTS/CTD; 100 controls (NA)	WES	[77]
		The R129G and c.-5A>G variants were not identified in any GTS/CTD individuals.	132 GTS/CTD (Han Chinese)	Genotyping	[65]
LHX6 (LIM homeobox 6)	9q33.2	Association between GTS and rs3808901.	222 GTS trios (European)	Association (TDT)	[93]
		Association between GTS and rs3750486.	162 GTS; 148 GTS trios; 128 controls (Southern + Eastern European)	Meta-analysis, TDT	[55]
SORCS3 (Sortilin related VPS10 domain containing receptor 3)	10q25.1	Association between GTS and rs9787523 {g}.	232,964 GTS/AN/ADHD/ASD/BP/ MD/OCD/SCZ (incl. 4,645 GTS (European, North American)); 494,162 controls (Mixed)	GWAS	[80]
		SORCS3 was associated with GTS, ADHD and ASD.	93,294 GTS/ADHD/ASD/OCD (Mixed); 51,311 controls (Mixed)	Cross-disorder GWAS meta-analysis	[90]
ADRA2A (Adrenoceptor alpha 2A)	10q25.2	No association found between GTS and the -1291 promoter region polymorphism.	113 nuclear GTS families (Mixed)	Linkage, Association (TDT)	[94]
		No association found between GTS and the C-1291G and C753G polymorphisms.	160 GTS children; 83 controls (Taiwanese)	Association	[95]
TH (Tyrosine hydroxylase)	11p15.6	No association found between GTS and the tetranucleotide repeat polymorphism.	5 GTS families (Canadian)	Linkage analysis	[35]
		No linkage found between GTS and *DBH*.	One 85 indv. pedigree with 29 GTS indv + 20 CMT indv (British)	Linkage analysis	[26]
DYNC2H1 (Dynein cytoplasmic 2 heavy chain 1)	11q22.3	Identification of *de novo* mutation.	9 GTS trios; 1 quartet with GTS CC MZ twins (NA)	WES	[96]
		DYNC2H1 was identified as a low confidence risk gene.	13 GTS multiplex families (Caucasian)	WES, genotyping	[44]
ROBO3 (Roundabout guidance receptor 3)	11q24.2	*ROBO3* was identified as a medium confidence risk gene.	13 GTS multiplex families (Caucasian)	WES, genotyping	[44]

Gene	Chromosome	Finding	Sample Size (Ancestry)	Analysis	Reference
		No association found between GTS and SNPs rs11219819, rs4936957, rs3923890, rs11219821, rs4606490, and rs3802905.	255 GTS children in 155 nuclear families (NA)	Association (TDT)	[97]
ROBO4 (Roundabout guidance receptor 4)	11q24.2	*ROBO4* variants identified in two GTS individuals.	120 GTS; 788 controls (French)	Sequence analysis	[7]
		No association found between GTS and SNPs rs4078483, rs4635093, rs6590109, and rs12823.	255 GTS children in 155 nuclear families (NA)	Association (TDT)	[97]
FLT3 (Fms related receptor tyrosine kinase 3)	13q12.2	Association between GTS and SNP rs2504235 in *FLT3*, although this association was not replicated.	4,819 GTS; 9,488 controls (European); Replication: 706 GTS; 466 with tic disorders; 6,068 controls (Icelandic)	GWAS meta-analysis	[98]
		The statistical significance of the lymphocyte gene set in GTS was driven partly by SNP rs1933437 in *FLT3.*	3,581 GTS (European incl. Ashkenazi Jewish and French Canadian (+Latin American)); 7,682 ancestry matched controls	Genome-wide pathway analysis	[81]
CHRNA7 (Cholinergic receptor nicotinic alpha 7 subunit)	15q13.3	Two microduplications (15q13.3 and Xq21.31) present in family; *CHRNA7* were within the 15q duplication.	One GTS family (NA)	Breakpoint mapping	[99]
		Association between GTS and comorbid OCD and promoter variant -86T.	206 GTS; 450 controls (Danish)	Association	[100]
GRIN2A (Glutamate ionotropic receptor NMDA type subunit 2A)	16p13.2	No association found between GTS and *GRIN2A.*	201 GTS (from families and singletons); 253 controls (USA); replication: 44 GTS (from families and singletons); 73 controls (Spain)	Genotyping, association (TDT)	[27]
		GRIN2A was identified as a high confidence risk gene.	13 GTS multiplex families (Caucasian)	WES, genotyping	[44]
SLC6A2 (Solute carrier family 6 member 2)	16q12.2	*SLC6A2* was identified as a low confidence risk gene.	13 GTS multiplex families (Caucasian)	WES, genotyping	[44]
		No association found between GTS and the T182C and G1287A polymorphisms.	115 GTS+ADHD; 110 GTS; 120 controls (Caucasian)	Association	[101]
CDH26 (Cadherin 26)	20q13.33	The statistical significance of the cell adhesion and transsynaptic signaling gene	3,581 GTS (European incl. Ashkenazi Jewish and French Canadian (+Latin American));	Genome-wide pathway analysis	[81]

Gene	Chromosome	Finding	Sample Size (Ancestry)	Analysis	Reference
		set in GTS was driven partly by *CDH26.* (MAGMA).	7,682 ancestry matched controls		
		The statistical significance of the cell adhesion and transsynaptic signaling gene set in GTS was driven partly by the SNP rs1002762 in *CDH26.* (Set-based association).	3,581 GTS (European incl. Ashkenazi Jewish and French Canadian (+Latin American)); 7,682 ancestry matched controls	Genome-wide pathway analysis	[81]
NCAM2 (Neural cell adhesion molecule 2)	21q21.1	The statistical significance of the cell adhesion and transsynaptic signaling gene set in GTS was driven partly by *NCAM2.* (MAGMA).	3,581 GTS (European incl. Ashkenazi Jewish and French Canadian (+Latin American)); 7,682 ancestry matched controls	Genome-wide pathway analysis	[81]
		The statistical significance of the cell adhesion and transsynaptic signaling gene set in GTS was driven partly by the SNP rs2826825 in *NCAM2.* (Set-based association).	3,581 GTS (European incl. Ashkenazi Jewish and French Canadian (+Latin American)); 7,682 ancestry matched controls	Genome-wide pathway analysis	[81]
HTR2C (5-Hydroxytryptamine receptor 2C)	Xq23	Nominally significant association between GTS and SNPs rs518147 and rs3813929.	87 GTS; 311 controls (Caucasian)	Association	[46]
		No association found between GTS and SNPs rs518147 and rs3813929.	110 GTS; 440 controls (Han Chinese)	Association	[65]
AGRN (Agrin)	1p36.33	*AGRN* was identified as a high confidence risk gene.	13 GTS multiplex families (Caucasian)	WES, genotyping	[44]
ENO1 (Enolase 1)	1p36.23	Association between GTS and rs301799 {hf}.	232,964 GTS/AN/ADHD/ASD/BP/ MD/OCD/SCZ (incl. 4,645 GTS (European, North American)); 494,162 controls (Mixed)	GWAS	[80]
ENO1-IT1 (ENO1 intronic transcript 1)	1p36.23	Association between GTS and rs301799 {hf}.	232,964 GTS/AN/ADHD/ASD/BP/ MD/OCD/SCZ (incl. 4,645 GTS (European, North American)); 494,162 controls (Mixed)	GWAS	[80]
ERRFI1 (ERBB receptor feedback inhibitor 1)	1p36.23	Association between GTS and rs301799 {ha, hf}.	232,964 GTS/AN/ADHD/ASD/BP/	GWAS	[80]

Gene	Chromosome	Finding	Sample Size (Ancestry)	Analysis	Reference
			MD/OCD/SCZ (incl. 4,645 GTS (European, North American)); 494,162 controls (Mixed)		
PARK7 (Parkinsonism associated deglycase)	1p36.23	Association between GTS and rs301799 {ha, hf}.	232,964 GTS/AN/ADHD/ASD/BP/ MD/OCD/SCZ (incl. 4,645 GTS (European, North American)); 494,162 controls (Mixed)	GWAS	[80]
RERE (Arginine-glutamic acid dipeptide repeats)	1p36.23	Association between GTS and rs301799 {g, ha, hf, tss}.	232,964 GTS/AN/ADHD/ASD/BP/ MD/OCD/SCZ (incl. 4,645 GTS (European, North American)); 494,162 controls (Mixed)	GWAS	[80]
SLC45A1 (Solute carrier family 45 member 1)	1p36.23	Association between GTS and rs301799 {ha, hf, tss}.	232,964 GTS/AN/ADHD/ASD/BP/ MD/OCD/SCZ (incl. 4,645 GTS (European, North American)); 494,162 controls (Mixed)	GWAS	[80]
EPHB2 (EPH receptor B2)	1p36.12	*EPHB2* was identified as a medium confidence risk gene.	13 GTS multiplex families (Caucasian)	WES, genotyping	[44]
DHDDS (Dehydrodolichyl diphosphate synthase subunit)	1p36.11	*DHDDS* was identified as a low confidence risk gene.	13 GTS multiplex families (Caucasian)	WES, genotyping	[44]
DLGAP3 (DLG associated protein 3)	1p34.3	Association between GTS and rs11264126 as well as haplotypes containing rs11264126 and rs12141243. Association did not remain significant after correction for multiple testing.	289 GTS trios (USA, Canada, UK, Netherlands)	Association (TDT)	[102]
LHX8 (LIM homeobox 8)	1p31.1	Association between GTS and SNPs rs729833, rs12732329, and rs941032.	222 GTS trios (European)	Association (TDT)	[93]
NEGR1 (Neuronal growth regulator 1)	1p31.1	Association between GTS and rs7531118 {hf}.	232,964 GTS/AN/ADHD/ASD/BP/ MD/OCD/SCZ (incl. 4,645 GTS (European, North American)); 494,162 controls (Mixed)	GWAS	[80]

Gene	Chromosome	Finding	Sample Size (Ancestry)	Analysis	Reference
SORT1 (Sortilin 1)	1p13.3	*SORT1* was identified as a medium confidence risk gene.	13 GTS multiplex families (Caucasian)	WES, genotyping	[44]
ASH1L (ASH1 like histone lysine methyltransferase)	1q22	Association between GTS and *ASH1L*.	100 GTS trios (NA); Replication: 524 GTS (NA); 2,822 controls (ExAC, East Asian)	WES, TDT	[103]
CD84 (CD84 molecule)	1q23.3	Identification of *de novo* mutation.	9 GTS trios; 1 quartet with GTS CC MZ twins (NA)	WES	[96]
TNN (Tenascin N)	1q25.1	*TNN* was identified as a high confidence risk gene.	13 GTS multiplex families (Caucasian)	WES, genotyping	[44]
ADORA1 (Adenosine A1 receptor)	1q32.1	Association between GTS and SNP rs2228079.	162 GTS; 270 controls (Polish)	Association	[104]
NR5A2 (Nuclear receptor subfamily 5 group A member 2)	1q32.1	Identification of *de novo* mutation.	9 GTS trios; 1 quartet with GTS CC MZ twins (NA)	WES	[96]
KCNK1 (Potassium two pore domain channel subfamily K member 1)	1q42.2	*KCNK1* was identified as a medium confidence risk gene.	13 GTS multiplex families (Caucasian)	WES, genotyping	[44]
AHCTF1 (AT-hook containing transcription factor 1)	1q44	Identification of *de novo* mutation.	9 GTS trios; 1 quartet with GTS CC MZ twins (NA)	WES	[96]
KIDINS220 (Kinase D interacting substrate 220)	2p25.1	*KIDINS220* was identified as a medium confidence risk gene.	13 GTS multiplex families (Caucasian)	WES, genotyping	[44]
MDH1 (Malate dehydrogenase 1)	2p15	*MDH1* was associated with GTS, ADHD and ASD.	93,294 GTS/ADHD/ASD/OCD (Mixed); 51,311 controls (Mixed)	Cross-disorder GWAS meta-analysis	[90]
WDPCP (WD repeat containing planar cell polarity effector)	2p15	*WDPCP* was associated with GTS, ADHD and ASD.	93,294 GTS/ADHD/ASD/OCD (Mixed); 51,311 controls (Mixed)	Cross-disorder GWAS meta-analysis	[90]
MEIS1 (Meis homeobox 1)	2p14	No association between GTS and SNPs rs12469063, and rs2300478.	298 GTS trios; 24 GTS indv.; 290 controls (French Canadian)	Association	[64]
CYP26B1 (Cytochrome P450 family 26 subfamily B member 1)	2p13.2	Identification of *de novo* mutation.	9 GTS trios; 1 quartet with GTS CC MZ twins (NA)	WES	[96]
MRPL35 (Mitochondrial ribosomal protein L35)	2p11.2	Identification of *de novo* mutation.	9 GTS trios; 1 quartet with GTS CC MZ twins (NA)	WES	[96]

Gene	Chromosome	Finding	Sample Size (Ancestry)	Analysis	Reference
SLC5A7 (Solute carrier family 5 member 7)	2q12.3	No association between GTS and SNPs rs1013940, rs2433718, and rs4676169.	401 GTS trios; 400 controls (Han Chinese)	Association (TDT, HRR)	[105]
IL1B (Interleukin 1 beta)	2q13	No association found between GTS and the polymorphisms in exon 5 and the promoter region.	159 GTS children; 175 controls (Taiwanese)	Association	[106]
IL1RN (Interleukin 1 receptor antagonist)	2q13	Association between GTS and the *IL1RN**1 allele.	159 GTS children; 175 controls (Taiwanese)	Association	[106]
CNTNAP5 (Contactin associated protein like 5)	2q14.3	*CNTNAP5* was identified as a medium confidence risk gene.	13 GTS multiplex families (Caucasian)	WES, genotyping	[44]
CASP8 (Caspase 8)	2q33.1	*CASP8* was identified as a high confidence risk gene.	13 GTS multiplex families (Caucasian)	WES, genotyping	[44]
TTN (Titin)	2q31.2	Missense variant identified in GTS individuals.	15 GTS trios (Chinese); 1,153 control trios (SSC)	WES	[69]
DNAH7 (dynein axonemal heavy chain 7)	2q32.3	*DNAH7* was identified as a high confidence risk gene.	13 GTS multiplex families (Caucasian)	WES, genotyping	[44]
NRP2 (Neuropilin 2)	2q33.3	*NRP2* variants identified in GTS individuals.	120 GTS; 788 controls (French)	Sequence analysis	[7]
NCL (Nucleolin)	2q37.1	*NCL* was identified as a medium confidence risk gene.	13 GTS multiplex families (Caucasian)	WES, genotyping	[44]
CNTN6 (Contactin 6)	3p26.3	Association between GTS and *CNTN6* duplications.	2,434 GTS; 4,093 controls (European)	Association, CNV analysis	[75]
TADA3 (Transcriptional adaptor 3)	3p25.3	*TADA3* was identified as a high confidence risk gene.	13 GTS multiplex families (Caucasian)	WES, genotyping	[44]
NEK10 (NIMA related kinase 10)	3p24.1	Identification of *de novo* missense mutation.	9 GTS trios; 1 quartet with GTS CC MZ twins (NA)	WES	[96]
CCDC36 (Coiled-coil domain containing 36)	3p21.31	*CCDC36* was associated with GTS, ADHD and ASD.	93,294 GTS/ADHD/ASD/OCD (Mixed); 51,311 controls (Mixed)	Cross-disorder GWAS meta-analysis	[90]
CCDC71 (Coiled-coil domain containing 71)	3p21.31	*CCDC71* was associated with GTS, ADHD and ASD.	93,294 GTS/ADHD/ASD/OCD (Mixed); 51,311 controls (Mixed)	Cross-disorder GWAS meta-analysis	[90]
NICN1 (Nicolin 1)	3p21.31	*NICN1* was associated with GTS, ADHD and ASD.	93,294 GTS/ADHD/ASD/OCD (Mixed); 51,311 controls (Mixed)	Cross-disorder GWAS meta-analysis	[90]

Gene	Chromosome	Finding	Sample Size (Ancestry)	Analysis	Reference
USP4 (Ubiquitin specific peptidase 4)	3p21.31	*USP4* was associated with GTS, ADHD and ASD.	93,294 GTS/ADHD/ASD/OCD (Mixed); 51,311 controls (Mixed)	Cross-disorder GWAS meta-analysis	[90]
DCAF1 (DDB1 and CUL4 associated factor 1)	3p21.2	*DCAF1* was identified as a medium confidence risk gene.	13 GTS multiplex families (Caucasian)	WES, genotyping	[44]
STAB1 (Stabilin 1)	3p21.1	*De novo* missense mutation identified in *STAB1* in GTS individual.	119 GTS+ASD children; 2,603 ASD children (SSC)	GSEA	[76]
ROBO1 (Roundabout guidance receptor 1)	3p12.3	Identification of *de novo* mutation.	9 GTS trios; 1 quartet with GTS CC MZ twins (NA)	WES	[96]
ROBO2 (Roundabout guidance receptor 2)	3p12.3	The statistical significance of the cell adhesion and transsynaptic signaling gene set in GTS was driven partly by the SNP rs6773575 in *ROBO2.*	3,581 GTS (European incl. Ashkenazi Jewish and French Canadian (+Latin American)); 7,682 ancestry matched controls	Genome-wide pathway analysis	[81]
COL8A1 (Collagen type VIII alpha 1 chain)	3q12.1	Association between GTS and *COL8A1* duplication.	210 GTS; 285 controls; 53 GTS (Latin American)	Association, CNV analysis	[74]
CD47 (CD47 molecule)	3q13.12	The statistical significance of the cell adhesion and transsynaptic signaling gene set in GTS was driven partly by *CD47.*	3,581 GTS (European incl. Ashkenazi Jewish and French Canadian (+Latin American)); 7,682 ancestry matched controls	Genome-wide pathway analysis	[81]
LSAMP (Limbic system-associated membrane protein)	3q13.31	The statistical significance of the cell adhesion and transsynaptic signaling gene set in GTS was driven partly by *LSAMP.*	3,581 GTS (European incl. Ashkenazi Jewish and French Canadian (+Latin American)); 7,682 ancestry matched controls	Genome-wide pathway analysis	[81]
ZBTB20 (Zinc finger and BTB domain containing 20)	3q13.31	Missense variant identified in GTS individuals.	15 GTS trios (Chinese); 1,153 control trios (SSC)	WES	[69]
IL12A (Interleukin 12A)	3q25.33	The statistical significance of the lymphocyte gene set in GTS was driven partly by SNP rs2243123 in *IL12A.*	3,581 GTS (European incl. Ashkenazi Jewish and French Canadian (+Latin American)); 7,682 ancestry matched controls	Genome-wide pathway analysis	[81]
TNIK (TRAF2 and NCK interacting kinase)	3q26.2-.31	Missense variant identified in GTS individuals.	15 GTS trios (Chinese); 1,153 control trios (SSC)	WES	[69]

Gene	Chromosome	Finding	Sample Size (Ancestry)	Analysis	Reference
CLCN2 (Chloride voltage-gated channel 2)	3q27.1	G161S was found to co-segregate with GTS and associated with GTS in replication cohort.	One family with 3 GTS indv.; Replication: 207 tic disorder indv. incl. 111 GTS; 489 controls (Han Chinese)	WES, association	[107]
IGF2BP2 (Insulin like growth factor 2 MRNA binding protein 2)	3q27.2	Identification of *de novo* mutation.	9 GTS trios; 1 quartet with GTS CC MZ twins (NA)	WES	[96]
OPA1 (OPA1 mitochondrial dynamin like GTPase)	3q29	*OPA1* was identified as a probable GTS risk gene.	802 GTS trios; 1,184 control quartets (Mixed)	WES	[68]
ADRA2C (Adrenoceptor alpha 2C)	4p16.3	No association found between GTS and the ADRA2(CA)n polymorphism.	160 GTS children; 83 controls (Taiwanese)	Association	[95]
GAK (Cycling G associated kinase)	4p16.3	*GAK* was identified as a high confidence risk gene.	13 GTS multiplex families (Caucasian)	WES, genotyping	[44]
HTRA3 (HtrA serine peptidase 3)	4p16.1	*HTRA3* was identified as a medium confidence risk gene.	13 GTS multiplex families (Caucasian)	WES, genotyping	[44]
KLB (Klotho beta)	4p14	LGD variant identified in GTS individuals.	15 GTS trios (Chinese); 1,153 control trios (SSC)	WES	[69]
DCAF4L1 (DDB1 and CUL4 associated factor 4 like 1)	4p13	Association between GTS and rs34215985 {tss}.	232,964 GTS/AN/ADHD/ASD/BP/ MD/OCD/SCZ (incl. 4,645 GTS (European, North American)); 494,162 controls (Mixed)	GWAS	[80]
LIMCH1 (LIM and calponin homology domains 1)	4p13	Association between GTS and rs34215985 {ha, hf}.	232,964 GTS/AN/ADHD/ASD/BP/ MD/OCD/SCZ (incl. 4,645 GTS (European, North American)); 494,162 controls (Mixed)	GWAS	[80]
PHOX2B (Paired like homeobox 2B)	4p13	Association between GTS and rs34215985 {hf}.	232,964 GTS/AN/ADHD/ASD/BP/ MD/OCD/SCZ (incl. 4,645 GTS (European, North American)); 494,162 controls (Mixed)	GWAS	[80]
SLC30A9 (Solute carrier family 30 member 9)	4p13	Association between GTS and rs34215985 {g, q}.	232,964 GTS/AN/ADHD/ASD/BP/	GWAS	[80]

Gene	Chromosome	Finding	Sample Size (Ancestry)	Analysis	Reference
			MD/OCD/SCZ (incl. 4,645 GTS (European, North American)); 494,162 controls (Mixed)		
UCHL1 (Ubiquitin C-terminal hydrolase L1)	4p13	Association between GTS and rs34215985 {ha, hf}.	232,964 GTS/AN/ADHD/ASD/BP/ MD/OCD/SCZ (incl. 4,645 GTS (European, North American)); 494,162 controls (Mixed)	GWAS	[80]
G3BP2 (G3BP stress granule assembly factor 2)	4q21.1	Identification of *de novo* mutation.	9 GTS trios; 1 quartet with GTS CC MZ twins (NA)	WES	[96]
STPG2 (Sperm tail PG-rich repeat containing 2)	4q22.3	Identification of *de novo* mutation.	9 GTS trios; 1 quartet with GTS CC MZ twins (NA)	WES	[96]
MANBA (Mannosidase beta)	4q24	*MANBA* was associated with GTS, ADHD and ASD.	93,294 GTS/ADHD/ASD/OCD (Mixed); 51,311 controls (Mixed)	Cross-disorder GWAS meta-analysis	[90]
CXXC4 (CXXC finger protein 4)	4q24	*CXXC4* was associated with GTS, ADHD and ASD.	93,294 GTS/ADHD/ASD/OCD (Mixed); 51,311 controls (Mixed)	Cross-disorder GWAS meta-analysis	[90]
PCDH10 (Protocadherin 10)	4q28.3	*PCDH10* variants identified in GTS individuals.	120 GTS; 788 controls (French)	Sequence analysis	[7]
GRIA2 (Glutamate ionotropic receptor AMPA type subunit 2)	4q32.1	No association found between GTS and *GRIA2.*	201 GTS (from families and singletons); 253 controls (USA); replication: 44 GTS (from families and singletons); 73 controls (Spain)	Genotyping, association (TDT)	[27]
ZDHHC11 (Zinc finger DHHC-type containing 11)	5p15.33	Missense variant identified in GTS individuals.	15 GTS trios (Chinese); 1,153 control trios (SSC)	WES	[69]
DNAH5 (Dynein axonemal heavy chain 5)	5p15.2	*DNAH5* was identified as a high confidence risk gene.	13 GTS multiplex families (Caucasian)	WES, genotyping	[44]
CDH9 (Cadherin 9)	5p14.1	The statistical significance of the cell adhesion and transsynaptic signaling gene set in GTS was driven partly by *CDH9.*	3,581 GTS (European incl. Ashkenazi Jewish and French Canadian (+Latin American)); 7,682 ancestry matched controls	Genome-wide pathway analysis	[81]

Gene	Chromosome	Finding	Sample Size (Ancestry)	Analysis	Reference
RICTOR (RPTOR independent companion of MTOR complex 2)	5p13.1	Identification of *de novo* missense mutation.	9 GTS trios; 1 quartet with GTS CC MZ twins (NA)	WES	[96]
FGF10 (Fibroblast growth factor 10)	5p12	No association between GTS and SNPs rs2290070, rs980510, and rs11743802.	1 large GTS family (Canadian); 241 GTS children in 171 nuclear families (NA)	Linkage, Association (TDT)	[79]
ISL1 (ISL LIM homeobox 1)	5q11.1	No association between GTS and SNPs rs10491412, rs11954894, and rs2288648.	1 large GTS family (Canadian); 241 GTS children in 171 nuclear families (NA)	Linkage, Association (TDT)	[79]
ITGA1 (Integrin subunit alpha 1)	5q11.2	No association between GTS and SNPs rs13188662, rs2406217, rs2860025, rs1391983, rs12110170, rs2126953, rs7732839, rs2456205, rs2406369, rs1478438, rs1478442, rs10513003, rs1421927, and rs2193968.	1 large GTS family (Canadian); 241 GTS children in 171 nuclear families (NA)	Linkage, Association (TDT)	[79]
CD180 (CD180 molecule)	5q12.3	The statistical significance of the lymphocyte gene set in GTS was driven partly by SNP rs2230525 in *CD180.*	3,581 GTS (European incl. Ashkenazi Jewish and French Canadian (+Latin American)); 7,682 ancestry matched controls	Genome-wide pathway analysis	[81]
ALDH7A1 (Aldehyde dehydrogenase 7 family member A1)	5q23.2	*ALDH7A1* was identified as a low confidence risk gene.	13 GTS multiplex families (Caucasian)	WES, genotyping	[44]
FBN2 (Fibrillin 2)	5q23.3	*FBN2* was identified as a probable GTS risk gene.	802 GTS trios; 1,184 control quartets (Mixed)	WES	[68]
MATR3 (Matrin 3)	5q31.2	Identification of *de novo* mutation.	9 GTS trios; 1 quartet with GTS CC MZ twins (NA)	WES	[96]
PCDH1 (Protocadherin 1)	5q31.3	The statistical significance of the cell adhesion and transsynaptic signaling gene set in GTS was driven partly by *PCDH1.*	3,581 GTS (European incl. Ashkenazi Jewish and French Canadian (+Latin American)); 7,682 ancestry matched controls	Genome-wide pathway analysis	[81]
PCDH12 (Protocadherin 12)	5q31.3	*PCDH12* was identified as a low confidence risk gene.	13 GTS multiplex families (Caucasian)	WES, genotyping	[44]
CAMK2A (Calcium/calmodulin	5q32	No association found between GTS and *CAMK2A.*	201 GTS (from families and singletons); 253 controls (USA);	Genotyping, association (TDT)	[27]

Gene	Chromosome	Finding	Sample Size (Ancestry)	Analysis	Reference
dependent protein kinase II alpha)			replication: 44 GTS (from families and singletons); 73 controls (Spain)		
PPP2R2B (Protein phosphatase 2 regulatory subunit Bbeta)	5q32	Association between GTS and rs4495234 {g}.	232,964 GTS/AN/ADHD/ASD/BP/ MD/OCD/SCZ (incl. 4,645 GTS (European, North American)); 494,162 controls (Mixed)	GWAS	[80]
GRIA1 (Glutamate ionotropic receptor AMPA type subunit 1)	5q33.2	No association found between GTS and *GRIA1.*	201 GTS (from families and singletons); 253 controls (USA); replication: 44 GTS (from families and singletons); 73 controls (Spain)	Genotyping, association (TDT)	[27]
GPLD1 (Glycosylphosphatidylinosito l specific phospholipase D1)	6p22.3	*GPLD1* was identified as a high confidence risk gene.	13 GTS multiplex families (Caucasian)	WES, genotyping	[44]
MOG (Myelin oligodendrocyte glycoprotein)	6p22.1	No association found between GTS and the three microsatellite polymorphisms MOGa, MOGb, and MOGc.	197 GTS trios (Chinese)	Linkage, association (TDT)	[108]
LY6G6F (Lymphocyte antigen 6 family member G6F)	6p21.33	*LY6G6F* was associated with GTS and OCD.	93,294 GTS/ADHD/ASD/OCD (Mixed); 51,311 controls (Mixed)	Cross-disorder GWAS meta-analysis	[90]
MEGT1 (Megakaryocyte-enhanced gene transcript 1 protein)	6p21.33	*MEGT1* was associated with GTS and OCD.	93,294 GTS/ADHD/ASD/OCD (Mixed); 51,311 controls (Mixed)	Cross-disorder GWAS meta-analysis	[90]
APOM (Apolipoprotein M)	6p21.33	*APOM* was associated with GTS and OCD.	93,294 GTS/ADHD/ASD/OCD (Mixed); 51,311 controls (Mixed)	Cross-disorder GWAS meta-analysis	[90]
TBC1D22B (TBC1 domain family member 22B)	6p21.2	Identification of *de novo* mutation.	9 GTS trios; 1 quartet with GTS CC MZ twins (NA)	WES	[96]
RIMS1 (Regulating synaptic membrane exocytosis 1)	6q13	*RIMS1* was identified as a high confidence risk gene.	13 GTS multiplex families (Caucasian)	WES, genotyping	[44]
GRM1 (Glutamate metabotropic receptor 1)	6q24.3	No association found between GTS and *GRM1.*	201 GTS (from families and singletons); 253 controls (USA); replication: 44 GTS (from	Genotyping, association (TDT)	[27]

Gene	Chromosome	Finding	Sample Size (Ancestry)	Analysis	Reference
			families and singletons); 73 controls (Spain)		
ESR1 (Estrogen receptor 1)	6q25.1	*ESR1* was identified as a high confidence risk gene.	13 GTS multiplex families (Caucasian)	WES, genotyping	[44]
OPRM1 (Opioid receptor mu 1)	6q25.2	*OPRM1* was identified as a high confidence risk gene.	13 GTS multiplex families (Caucasian)	WES, genotyping	[44]
SYNJ2 (Synaptojanin 2)	6q25.3	*SYNJ2* was identified as a medium confidence risk gene.	13 GTS multiplex families (Caucasian)	WES, genotyping	[44]
HDAC9 (Histone deacetylase 9)	7p21.1	The statistical significance of the lymphocyte gene set in GTS was driven partly by SNP rs3801983 in *HDAC9.*	3,581 GTS (European incl. Ashkenazi Jewish and French Canadian (+Latin American)); 7,682 ancestry matched controls	Genome-wide pathway analysis	[81]
DNAH11 (Dynein axonemal heavy chain 11)	7p15.3	*DNAH11* was identified as a low confidence risk gene.	13 GTS multiplex families (Caucasian)	WES, genotyping	[44]
AEBP1 (AE binding protein 1)	7p13	*AEBP1* was identified as a low confidence risk gene.	13 GTS multiplex families (Caucasian)	WES, genotyping	[44]
VWC2 (von Willebrand factor C domain containing 2)	7p12.2	*VWC2* was identified as a medium confidence risk gene.	13 GTS multiplex families (Caucasian)	WES, genotyping	[44]
CALN1 (Calneuron 1)	7q11.22	*CALN1* was associated with GTS, ADHD and ASD.	93,294 GTS/ADHD/ASD/OCD (Mixed); 51,311 controls (Mixed)	Cross-disorder GWAS meta-analysis	[90]
ELN (Elastin)	7q11.23	Identification of *de novo* mutation.	9 GTS trios; 1 quartet with GTS CC MZ twins (NA)	WES	[96]
AKAP9 (A-kinase anchoring protein 9)	7q21.2	*AKAP9* was identified as a medium confidence risk gene.	13 GTS multiplex families (Caucasian)	WES, genotyping	[44]
CLDN15 (Claudin 15)	7q22.1	Identification of *de novo* mutation.	9 GTS trios; 1 quartet with GTS CC MZ twins (NA)	WES	[96]
VGF (VGF nerve growth factor inducible)	7q22.1	*VGF* was identified as a high confidence risk gene.	13 GTS multiplex families (Caucasian)	WES, genotyping	[44]
NRCAM (Neuronal cell adhesion molecule)	7q31.1	*NRCAM* was identified as a low confidence risk gene.	13 GTS multiplex families (Caucasian)	WES, genotyping	[44]
CADPS2 (Calcium dependent secretion activator 2)	7q31.32	*CADPS2* was identified as a high confidence risk gene.	13 GTS multiplex families (Caucasian)	WES, genotyping	[44]

Gene	Chromosome	Finding	Sample Size (Ancestry)	Analysis	Reference
GPR37 (G protein-coupled receptor 37)	7q31.33	*GPR37* was identified as a medium confidence risk gene.	13 GTS multiplex families (Caucasian)	WES, genotyping	[44]
STRIP2 (Striatin interacting protein 2)	7q32.1	Identification of *de novo* missense mutation.	9 GTS trios; 1 quartet with GTS CC MZ twins (NA)	WES	[96]
MGAM (Maltase-glucoamylase)	7q34	Missense variant identified in GTS individuals.	15 GTS trios (Chinese); 1,153 control trios (SSC)	WES	[69]
KMT2C (Lysine methyltransferase)	7q36.1	Missense variant identified in GTS individuals.	15 GTS trios (Chinese); 1,153 control trios (SSC)	WES	[69]
DPP6 (Dipeptidyl peptidase like 6)	7q36.2	Microdeletion identified in two GTS individuals.	Family with 3 GTS indv. (Italian)	CNV analysis	[109]
CSMD1 (CUB and sushi multiple domains 1)	8p23.2	Association between GTS and rs10108980 {g}.	232,964 GTS/AN/ADHD/ASD/BP/ MD/OCD/SCZ (incl. 4,645 GTS (European, North American)); 494,162 controls (Mixed)	GWAS	[80]
FAM167A (Family with sequence similarity 167 member A)	8p23.1	Identification of *de novo* mutation.	9 GTS trios; 1 quartet with GTS CC MZ twins (NA)	WES	[96]
ADRA1A (Adrenoceptor alpha 1A)	8p21.2	No association found between GTS and the Cys492Arg polymorphism.	113 nuclear GTS families (Mixed)	Linkage, Association (TDT)	[94]
OPRK1 (Opioid receptor kappa 1)	8q11.23	Association between GTS and variants in *OPRK1*.	120 GTS; 788 controls (French)	Sequence analysis	[7]
CBFA2T1 (RUNX1 partner transcriptional co-repressor 1)	8q21.3	*CBFA2T1* found in one of the translocation breakpoints, but no *CBFA2T1* mutations were identified in GTS individuals.	One multiplex GTS/tic family with [t(1;8)(q21.1;q22.1)]; 37 GTS (Mixed)	Breakpoint mapping, Sequence analysis	[110]
SLC1A1 (Solute carrier family 1 member 1)	9p24.2	Nominally significant association between GTS and the G allele of rs17812372.	465 GTS/CTD probands in 412 families (USA, Europe, South Korea); Replication: 1,285 GTS; Replication: 4,964 controls (European, Ashkenazi Jewish and French Canadian)	Association (TDT)	[73]
VPS13A (Vacuolar protein sorting 13 homolog A)	9q21.2	*VPS13A* was identified as a medium confidence risk gene.	13 GTS multiplex families (Caucasian)	WES, genotyping	[44]
NTRK2 (Neurotrophic receptor tyrosine kinase 2)	9q21.33	No association found between GTS and *NTRK2.*	201 GTS (from families and singletons); 253 controls (USA); replication: 44 GTS (from	Genotyping, association (TDT)	[27]

Gene	Chromosome	Finding	Sample Size (Ancestry)	Analysis	Reference
			families and singletons); 73 controls (Spain)		
GABBR2 (Gamma-aminobutyric acid type B receptor subunit 2)	9q22.33	The statistical significance of the ligand-gated ion channel signaling gene set in GTS was driven partly by SNPs rs2259639 and rs1930415 in *GABBR2.*	3,581 GTS (European incl. Ashkenazi Jewish and French Canadian (+Latin American)); 7,682 ancestry matched controls	Genome-wide pathway analysis	[81]
GRIN3A (Glutamate ionotropic receptor NMDA type subunit 3A)	9q31.1	No association found between GTS and *GRIN3A.*	201 GTS (from families and singletons); 253 controls (USA); replication: 44 GTS (from families and singletons); 73 controls (Spain)	Genotyping, association (TDT)	[27]
COL27A1 (Collagen type XXVII alpha 1 chain)	9q32	No markers reached a genome-wide threshold of significance, but rs7868992 in *COL27A1* was the top signal in both cohorts.	1,285 GTS; 4,964 controls (European, Ashkenazi Jewish, and French Canadian); Replication: 211 GTS; 285 controls (Latin American)	GWAS	[111]
GRIN1 (Glutamate ionotropic receptor NMDA type subunit 1)	9q34.3	No association found between GTS and *GRIN1.*	201 GTS (from families and singletons); 253 controls (USA); replication: 44 GTS (from families and singletons); 73 controls (Spain)	Genotyping, association (TDT)	[27]
OLFM1 (Olfactomedin 1)	9q34.3	The translocation breakpoints were shown to disrupt *OLFM1*, but no *OLFM1* variants found in the GTS cohort.	1 GTS/OCD/ADHD indv. with [t(3;9)(q25.1;q34.3)]; 175 GTS (Danish)	Breakpoint mapping, sequence analysis	[112]
RAB18 (Ras-related protein Rab-18)	10p12.1	Missense variant identified in GTS individuals.	15 GTS trios (Chinese); 1,153 control trios (SSC)	WES	[69]
ANK3 (Ankyrin 3)	10q21.2	*ANK3* was identified as a high confidence risk gene.	13 GTS multiplex families (Caucasian)	WES, genotyping	[44]
CTNNA3 (Catenin Alpha 3)	10q21.3	Association between GTS and *CTNNA3* deletions.	111 GTS; 73 controls (Caucasian)	Association, CNV analysis	[51]
RUFY2 (RUN and FYVE domain containing 2)	10q21.3	*De novo* missense mutation identified in *RUFY2* in GTS individual.	119 GTS+ASD children; 2,603 ASD children (SSC)	GSEA	[76]
CDH23 (Cadherin related 23)	10q22.1	*CDH23* was identified as a high confidence risk gene.	13 GTS multiplex families (Caucasian)	WES, genotyping	[44]

Gene	Chromosome	Finding	Sample Size (Ancestry)	Analysis	Reference
CDHR1 (Cadherin related family member 1)	10q23.1	*CDHR1* was identified as a high confidence risk gene.	13 GTS multiplex families (Caucasian)	WES, genotyping	[44]
ANO9 (Anoctamin 9)	11p15.5	Identification of *de novo* mutation.	9 GTS trios; 1 quartet with GTS CC MZ twins (NA)	WES	[96]
MUC2 (Mucin 2, oligomeric mucus/gel-forming)	11p15.5	Missense variant identified in GTS individuals.	15 GTS trios (Chinese); 1,153 control trios (SSC)	WES	[69]
PPFIBP2 (PPFIA binding protein 2)	11p15.4	Identification of *de novo* mutation.	9 GTS trios; 1 quartet with GTS CC MZ twins (NA)	WES	[96]
ADM (Adrenomedullin)	11p15.4	*ADM* was identified as a medium confidence risk gene.	13 GTS multiplex families (Caucasian)	WES, genotyping	[44]
GTF2H1 (General transcription factor IIH subunit 1)	11p15.1	Missense variant identified in GTS individuals.	15 GTS trios (Chinese); 1,153 control trios (SSC)	WES	[69]
BDNF (Brain derived neurotrophic factor)	11p14.1	No association found between GTS and *BDNF.*	201 GTS (from families and singletons); 253 controls (USA); replication: 44 GTS (from families and singletons); 73 controls (Spain)	Genotyping, association (TDT)	[27]
AMBRA1 (Autophagy and beclin 1 regulator 1)	11p11.2	*De novo* missense mutation identified in *AMBRA1* in GTS individual.	119 GTS+ASD children; 2,603 ASD children (SSC)	GSEA	[76]
CAPN1 (Calpain 1)	11q13.1	Identification of *de novo* mutation.	9 GTS trios; 1 quartet with GTS CC MZ twins (NA)	WES	[96]
CTTN (Cortactin)	11q13.3	The statistical significance of the cell adhesion and transsynaptic signaling gene set in GTS was driven partly by *CTTN.*	3,581 GTS (European incl. Ashkenazi Jewish and French Canadian (+Latin American)); 7,682 ancestry matched controls	Genome-wide pathway analysis	[81]
PICALM (Phosphatidylinositol binding clathrin assembly protein)	11q14.2	Nominally significant association between GTS and the A allele of rs621942.	465 GTS/CTD probands in 412 families (USA, Europe, South Korea); Replication: 1,285 GTS; Replication: 4,964 controls (European, Ashkenazi Jewish and French Canadian)	Association (TDT)	[73]
GRM5 (Glutamate metabotropic receptor 5)	11q14.3	No association found between GTS and *GRM5.*	201 GTS (from families and singletons); 253 controls (USA); replication: 44 GTS (from	Genotyping, association (TDT)	[27]

Gene	Chromosome	Finding	Sample Size (Ancestry)	Analysis	Reference
			families and singletons); 73 controls (Spain)		
NAALAD2 (N-acetylated alpha-linked acidic dipeptidase 2)	11q14.3	*NAALAD2* was identified as a low confidence risk gene.	13 GTS multiplex families (Caucasian)	WES, genotyping	[44]
TYR (Tyrosinase)	11q14.3	No linkage found between GTS and *TYR*.	One 85 indv. pedigree with 29 GTS indv + 20 CMT indv (British)	Linkage analysis	[26]
GRIA4 (Glutamate ionotropic receptor AMPA type subunit 4)	11q22.3	No association found between GTS and *GRIA4.*	201 GTS (from families and singletons); 253 controls (USA); replication: 44 GTS (from families and singletons); 73 controls (Spain)	Genotyping, association (TDT)	[27]
IL10RA (Interleukin 10 receptor subunit alpha)	11q23	No association found between GTS and SNPs rs3135932 and rs2229113.	77 GTS (Austrian); 260 controls (NA)	Association	[113]
HTR3A (5-hydroxytryptamine receptor 3A)	11q23.2	Identification of five sequence variants, not expected to be associated with GTS.	49 GTS; 47 controls (NA)	Sequence analysis	[114]
GRIK4 (Glutamate ionotropic receptor kainite type subunit 4)	11q23.3	The statistical significance of the ligand-gated ion channel signaling gene set in GTS was driven partly by SNP rs949054 in *GRIK4.*	3,581 GTS (European incl. Ashkenazi Jewish and French Canadian (+Latin American)); 7,682 ancestry matched controls	Genome-wide pathway analysis	[81]
HTR3B (5-hydroxytryptamine receptor 3B)	11q23.3	Identification of six sequence variants, not expected to be associated with GTS.	49 GTS; 47 controls (NA)	Sequence analysis	[114]
ETS1 (ETS proto-oncogene 1, transcription factor)	11q24.3	*ETS1* was identified as a high confidence risk gene.	13 GTS multiplex families (Caucasian)	WES, genotyping	[44]
NTM (Neurotrimin)	11q25	The statistical significance of the cell adhesion and transsynaptic signaling gene set in GTS was driven partly by the SNPs rs7925725 and rs12224080 in *NTM.*	3,581 GTS (European incl. Ashkenazi Jewish and French Canadian (+Latin American)); 7,682 ancestry matched controls	Genome-wide pathway analysis	[81]
OPCML (Opioid binding protein/cell adhesion molecule like)	11q25	The statistical significance of the cell adhesion and transsynaptic signaling gene set in GTS was driven partly by *OPCML.*	3,581 GTS (European incl. Ashkenazi Jewish and French Canadian (+Latin American));	Genome-wide pathway analysis	[81]

Gene	Chromosome	Finding	Sample Size (Ancestry)	Analysis	Reference
			7,682 ancestry matched controls		
CLEC12A (C-type lectin domain family 12 member A)	12p13.2	Identification of *de novo* mutation.	9 GTS trios; 1 quartet with GTS CC MZ twins (NA)	WES	[96]
GRIN2B (Glutamate ionotropic receptor NMDA type subunit 2B)	12p13.1	No association found between GTS and *GRIN2B.*	201 GTS (from families and singletons); 253 controls (USA); replication: 44 GTS (from families and singletons); 73 controls (Spain)	Genotyping, association (TDT)	[27]
NCKAP5L (NCK associated protein 5 like)	12q13.12	*NCKAP5L* was identified as a medium confidence risk gene.	13 GTS multiplex families (Caucasian)	WES, genotyping	[44]
ZDHHC17 (Zinc finger DHHC-type containing 17)	12q21.2	*ZDHHC17* was identified as a high confidence risk gene.	13 GTS multiplex families (Caucasian)	WES, genotyping	[44]
NOS1 (Nitric oxide synthase 1)	12q24.22	*NOS1* variants identified in GTS individuals.	120 GTS; 788 controls (French)	Sequence analysis	[7]
CENPJ (Centromere protein J)	13q12.12	*CENPJ* was identified as a high confidence risk gene.	13 GTS multiplex families (Caucasian)	WES, genotyping	[44]
KATNAL1 (Katanin catalytic subunit A1 like 1)	13q12.3	Identification of *de novo* mutation.	9 GTS trios; 1 quartet with GTS CC MZ twins (NA)	WES	[96]
HTR2A (5-hydroxytryptamine receptor 2A)	13q14.2	No association found between GTS and the T102C, A1438G, and His452Tyr polymorphisms.	87 GTS; 311 controls (Caucasian)	Association	[46]
SLITRK6 (SLIT and NTRK like family member 6)	13q31.1	No association between GTS and SNPs rs9513670, rs3825413, and rs7336083.	399 GTS trios (Han Chinese)	Association (TDT, HRR)	[115]
SLITRK5 (SLIT and NTRK like family member 5)	13q31.2	No association between GTS and SNPs rs4640042, rs9582391, and rs868919.	303 families incl. 377 GTS children (Mostly Caucasian)	Association (TDT)	[116]
COL4A2 (Collagen type IV alpha 2 chain)	13q34	*COL4A2* was identified as a medium confidence risk gene.	13 GTS multiplex families (Caucasian)	WES, genotyping	[44]
NPAS3 (Neuronal PAS domain protein 3)	14q13.1	Association between GTS and rs36063234 {g, tss}.	232,964 GTS/AN/ADHD/ASD/BP/ MD/OCD/SCZ (incl. 4,645 GTS (European, North American)); 494,162 controls (Mixed)	GWAS	[80]
FSCB (Fibrous sheath CABYR binding protein)	14q21.2	Association between GTS and *FSCB* deletions.	111 GTS; 73 controls (Caucasian)	Association, CNV analysis	[51]

Gene	Chromosome	Finding	Sample Size (Ancestry)	Analysis	Reference
KCNH5 (Potassium voltage-gated channel subfamily H member 5)	14q23.2	*KCNH5* was identified as a high confidence risk gene.	13 GTS multiplex families (Caucasian)	WES, genotyping	[44]
NDUFB1 (NADH:ubiquinone oxidoreductase subunit B1)	14q32.12	*NDUFB1* was identified as a medium confidence risk gene.	13 GTS multiplex families (Caucasian)	WES, genotyping	[44]
SLC25A29 (Solute carrier family 25 member 29)	14q32.2	Identification of *de novo* mutation.	9 GTS trios; 1 quartet with GTS CC MZ twins (NA)	WES	[96]
HSP90AA1 (Heat shock protein 90 alpha family class A member 1)	14q32.31	LGD variant identified in GTS individuals.	15 GTS trios (Chinese); 1,153 control trios (SSC)	WES	[69]
APOPT1 (Cytochrome C oxidase assembly factor)	14q32.33	Association between GTS and rs10149470 {fg}.	232,964 GTS/AN/ADHD/ASD/BP/ MD/OCD/SCZ (incl. 4,645 GTS (European, North American)); 494,162 controls (Mixed)	GWAS	[80]
C14orf2 (ATP synthase membrane subunit 6.8PL)	14q32.33	Association between GTS and rs10149470 {ha}.	232,964 GTS/AN/ADHD/ASD/BP/ MD/OCD/SCZ (incl. 4,645 GTS (European, North American)); 494,162 controls (Mixed)	GWAS	[80]
CKB (Creatine kinase B)	14q32.33	Association between GTS and rs10149470 {ha, hf}.	232,964 GTS/AN/ADHD/ASD/BP/ MD/OCD/SCZ (incl. 4,645 GTS (European, North American)); 494,162 controls (Mixed)	GWAS	[80]
EXOC3L4 (Exocyst complex component 3 like 4)	14q32.33	Association between GTS and rs10149470 {hf}.	232,964 GTS/AN/ADHD/ASD/BP/ MD/OCD/SCZ (incl. 4,645 GTS (European, North American)); 494,162 controls (Mixed)	GWAS	[80]
KLC1 (Kinesin light chain 1)	14q32.33	Association between GTS and rs10149470 {ha}.	232,964 GTS/AN/ADHD/ASD/BP/ MD/OCD/SCZ (incl. 4,645 GTS (European, North American)); 494,162 controls (Mixed)	GWAS	[80]

Gene	Chromosome	Finding	Sample Size (Ancestry)	Analysis	Reference
LINC00677 (Long intergenic non-protein coding RNA 677)	14q32.33	Association between GTS and rs10149470 {hf}.	232,964 GTS/AN/ADHD/ASD/BP/ MD/OCD/SCZ (incl. 4,645 GTS (European, North American)); 494,162 controls (Mixed)	GWAS	[80]
MARK3 (Microtubule affinity regulating kinase 3)	14q32.33	Association between GTS and rs10149470 {ha}.	232,964 GTS/AN/ADHD/ASD/BP/ MD/OCD/SCZ (incl. 4,645 GTS (European, North American)); 494,162 controls (Mixed)	GWAS	[80]
TNFAIP2 (TNF alpha induced protein 2)	14q32.33	Association between GTS and rs10149470 {hf}.	232,964 GTS/AN/ADHD/ASD/BP/ MD/OCD/SCZ (incl. 4,645 GTS (European, North American)); 494,162 controls (Mixed)	GWAS	[80]
TRAF3 (TNF receptor associated factor 3)	14q32.33	Association between GTS and rs10149470 {ha}.	232,964 GTS/AN/ADHD/ASD/BP/ MD/OCD/SCZ (incl. 4,645 GTS (European, North American)); 494,162 controls (Mixed)	GWAS	[80]
TRMT61A (TRNA methyltransferase 61A)	14q32.33	Association between GTS and rs10149470 {ha}.	232,964 GTS/AN/ADHD/ASD/BP/ MD/OCD/SCZ (incl. 4,645 GTS (European, North American)); 494,162 controls (Mixed)	GWAS	[80]
NIPA1 (NIPA magnesium transporter 1)	15q11.2	*NIPA1* was identified as a medium confidence risk gene.	13 GTS multiplex families (Caucasian)	WES, genotyping	[44]
TUBGCP5 (Tubulin gamma complex associated protein 5)	15q11.2	*TUBGCP5* was identified as a high confidence risk gene.	13 GTS multiplex families (Caucasian)	WES, genotyping	[44]
GABRB3 (Gamma-aminobutyric acid type A receptor beta3 subunit)	15q12	*GABRB3* was identified as a high confidence risk gene.	13 GTS multiplex families (Caucasian)	WES, genotyping	[44]
C15orf53 (Long intergenic non-protein coding RNA 2694)	15q14	Association between GTS and rs12898460 {tss}.	232,964 GTS/AN/ADHD/ASD/BP/	GWAS	[80]

Gene	Chromosome	Finding	Sample Size (Ancestry)	Analysis	Reference
			MD/OCD/SCZ (incl. 4,645 GTS (European, North American)); 494,162 controls (Mixed)		
FAM98B (Family with sequence similarity 98 member B)	15q14	Association between GTS and rs12898460 {ha, hf}.	232,964 GTS/AN/ADHD/ASD/BP/ MD/OCD/SCZ (incl. 4,645 GTS (European, North American)); 494,162 controls (Mixed)	GWAS	[80]
FSIP1 (Fibrous sheath interacting protein 1)	15q14	Association between GTS and rs12898460 {hf}.	232,964 GTS/AN/ADHD/ASD/BP/ MD/OCD/SCZ (incl. 4,645 GTS (European, North American)); 494,162 controls (Mixed)	GWAS	[80]
RASGRP1 (RAS guanyl releasing protein 1)	15q14	Association between GTS and rs12898460 {ha, hf}.	232,964 GTS/AN/ADHD/ASD/BP/ MD/OCD/SCZ (incl. 4,645 GTS (European, North American)); 494,162 controls (Mixed)	GWAS	[80]
THBS1 (Thrombospondin 1)	15q14	Association between GTS and rs12898460 {hf}.	232,964 GTS/AN/ADHD/ASD/BP/ MD/OCD/SCZ (incl. 4,645 GTS (European, North American)); 494,162 controls (Mixed)	GWAS	[80]
STARD9 (StAR related lipid transfer domain containing 9)	15q15.2	*STARD9* was identified as a low confidence risk gene.	13 GTS multiplex families (Caucasian)	WES, genotyping	[44]
UNC13C (Unc-13 homolog C)	15q21.3	*UNC13C* was identified as a high confidence risk gene.	13 GTS multiplex families (Caucasian)	WES, genotyping	[44]
SMAD6 (SMAD family member 6)	15q22.31	Identification of *de novo* mutation.	9 GTS trios; 1 quartet with GTS CC MZ twins (NA)	WES	[96]
MAP2K5 (Mitogen-activated protein kinase kinase 5)	15q23	No association between GTS and SNPs rs12593813, rs11635424, rs884202, rs4489954, rs3784709, rs1026732, and rs6494696.	298 GTS trios; 24 GTS indv.; 290 controls (French Canadian)	Association	[64]
RAB11FIP3 (RAB11 family interacting protein 3)	16p13.3	*RAB11FIP3* was identified as a high confidence risk gene.	13 GTS multiplex families (Caucasian)	WES, genotyping	[44]

Gene	Chromosome	Finding	Sample Size (Ancestry)	Analysis	Reference
RBFOX1 (RNA binding fox-1 homolog 1)	16p13.3	Association between GTS and rs7193263 {g}.	232,964 GTS/AN/ADHD/ASD/BP/ MD/OCD/SCZ (incl. 4,645 GTS (European, North American)); 494,162 controls (Mixed)	GWAS	[80]
ABCC1 (ATP binding cassette subfamily C member 1)	16p13.11	*ABCC1* was identified as a low confidence risk gene.	13 GTS multiplex families (Caucasian)	WES, genotyping	[44]
DNAH3 (Dynein axonemal heavy chain 3)	16p12.3	*DNAH3* was identified as a high confidence risk gene.	13 GTS multiplex families (Caucasian)	WES, genotyping	[44]
SYT17 (Synaptotagmin 17)	16p12.3	*SYT17* was identified as a high confidence risk gene.	13 GTS multiplex families (Caucasian)	WES, genotyping	[44]
TNRC6A (Trinucleotide repeat containing adaptor 6A)	16p12.1	Identification of *de novo* missense mutation.	9 GTS trios; 1 quartet with GTS CC MZ twins (NA)	WES	[96]
MAPK3 (Mitogen-activated protein kinase 3)	16p11.2	*MAPK3* was identified as a low confidence risk gene.	13 GTS multiplex families (Caucasian)	WES, genotyping	[44]
CDH5 (Cadherin 5)	16q21	The statistical significance of the cell adhesion and transsynaptic signaling gene set in GTS was driven partly by *CDH5.*	3,581 GTS (European incl. Ashkenazi Jewish and French Canadian (+Latin American)); 7,682 ancestry matched controls	Genome-wide pathway analysis	[81]
CX3CL1 (C-X3-C motif chemokine ligand 1)	16q21	*CX3CL1* was identified as a high confidence risk gene.	13 GTS multiplex families (Caucasian)	WES, genotyping	[44]
NOL3 (Nucleolar protein 3)	16q22.1	Missense variant identified in GTS individuals.	15 GTS trios (Chinese); 1,153 control trios (SSC)	WES	[69]
MAP1LC3B (Microtubule associated protein 1 light chain 3 beta)	16q24.2	*MAP1LC3B* was identified as a high confidence risk gene.	13 GTS multiplex families (Caucasian)	WES, genotyping	[44]
C1QBP (Complement C1q binding protein)	17p13.2	The statistical significance of the cell adhesion and transsynaptic signaling gene set in GTS was driven partly by *C1QBP.*	3,581 GTS (European incl. Ashkenazi Jewish and French Canadian (+Latin American)); 7,682 ancestry matched controls	Genome-wide pathway analysis	[81]
WNT3 (Wnt family member 3)	17q21.31	*WNT3* was associated with GTS, ADHD and ASD.	93,294 GTS/ADHD/ASD/OCD (Mixed); 51,311 controls (Mixed)	Cross-disorder GWAS meta-analysis	[90]

Gene	Chromosome	Finding	Sample Size (Ancestry)	Analysis	Reference
KANSL1 (KAT8 regulatory NSL complex subunit 1)	17q21.31	*KANSL1* was associated with GTS, ADHD and ASD.	93,294 GTS/ADHD/ASD/OCD (Mixed); 51,311 controls (Mixed)	Cross-disorder GWAS meta-analysis	[90]
CRHR1 (Corticotropin releasing hormone receptor 1)	17q21.31	*CRHR1* was associated with GTS, ADHD and ASD.	93,294 GTS/ADHD/ASD/OCD (Mixed); 51,311 controls (Mixed)	Cross-disorder GWAS meta-analysis	[90]
MAPT (Microtubule associated protein tau)	17q21.31	*MAPT* was associated with GTS, ADHD and ASD.	93,294 GTS/ADHD/ASD/OCD (Mixed); 51,311 controls (Mixed)	Cross-disorder GWAS meta-analysis	[90]
IGF2BP1 (Insulin like growth factor 2 mRNA binding protein 1)	17q21.32	*IGF2BP1* was identified as a high confidence risk gene.	13 GTS multiplex families (Caucasian)	WES, genotyping	[44]
GRIN2C (Glutamate ionotropic receptor NMDA type subunit 2C)	17q25.1	No association found between GTS and *GRIN2C.*	201 GTS (from families and singletons); 253 controls (USA); replication: 44 GTS (from families and singletons); 73 controls (Spain)	Genotyping, association (TDT)	[27]
TBCD (Tubulin folding cofactor D)	17q25.3	Nominally significant association between GTS and the G allele of rs3744161.	465 GTS/CTD probands in 412 families (USA, Europe, South Korea); Replication: 1,285 GTS; Replication: 4,964 controls (European, Ashkenazi Jewish and French Canadian)	Association (TDT)	[73]
DCC (DCC Netrin 1 receptor)	18q21.2	Association between GTS and rs8084351 {g, q}.	232,964 GTS/AN/ADHD/ASD/BP/ MD/OCD/SCZ (incl. 4,645 GTS (European, North American)); 494,162 controls (Mixed)	GWAS	[80]
MBP (Maltose-binding protein)	18q23	The statistical significance of the cell adhesion and transsynaptic signaling gene set in GTS was driven partly by *MBP.*	3,581 GTS (European incl. Ashkenazi Jewish and French Canadian (+Latin American)); 7,682 ancestry matched controls	Genome-wide pathway analysis	[81]
GRIN3B (Glutamate ionotropic receptor NMDA type subunit 3B)	19p13.3	No association found between GTS and *GRIN3B.*	201 GTS (from families and singletons); 253 controls (USA); replication: 44 GTS (from	Genotyping, association (TDT)	[27]

Gene	Chromosome	Finding	Sample Size (Ancestry)	Analysis	Reference
			families and singletons); 73 controls (Spain)		
ABCA7 (ATP binding cassette subfamily A member 7)	19p13.3	*ABCA7* was identified as a medium confidence risk gene.	13 GTS multiplex families (Caucasian)	WES, genotyping	[44]
SUGP1 (SURP and G-patch domain containing 1)	19p13.11	*SUGP1* was identified as a low confidence risk gene.	13 GTS multiplex families (Caucasian)	WES, genotyping	[44]
CADM4 (Cell adhesion molecule 4)	19q13.31	The statistical significance of the cell adhesion and transsynaptic signaling gene set in GTS was driven partly by *CADM4.*	3,581 GTS (European incl. Ashkenazi Jewish and French Canadian (+Latin American)); 7,682 ancestry matched controls	Genome-wide pathway analysis	[81]
GRIN2D (Glutamate ionotropic receptor NMDA type subunit 2D)	19q13.33	No association found between GTS and *GRIN2D.*	201 GTS (from families and singletons); 253 controls (USA); replication: 44 GTS (from families and singletons); 73 controls (Spain)	Genotyping, association (TDT)	[27]
SHANK1 (SH3 and multiple ankyrin repeat domains 1)	19q13.33	*SHANK1* variants identified in GTS individuals.	120 GTS; 788 controls (French)	Sequence analysis	[7]
NCR1 (Natural cytotoxicity triggering receptor 1)	19q13.42	The statistical significance of the lymphocyte gene set in GTS was driven partly by SNP rs16986092 near *NCR1.*	3,581 GTS (European incl. Ashkenazi Jewish and French Canadian (+Latin American)); 7,682 ancestry matched controls	Genome-wide pathway analysis	[81]
NLRP7 (NLR family pyrin domain containing 7)	19q13.42	The statistical significance of the lymphocyte gene set in GTS was driven partly by SNP rs16986092 near *NLRP.*	3,581 GTS (European incl. Ashkenazi Jewish and French Canadian (+Latin American)); 7,682 ancestry matched controls	Genome-wide pathway analysis	[81]
XRN2 (5’-3’ exoribonuclease 2)	20p11.23-p11.24	*XRN2* was associated with GTS, ADHD and ASD.	93,294 GTS/ADHD/ASD/OCD (Mixed); 51,311 controls (Mixed)	Cross-disorder GWAS meta-analysis	[90]
APMAP (Adipocyte plasma membrane associated protein)	20p11.21	Missense variant identified in GTS individuals.	15 GTS trios (Chinese); 1,153 control trios (SSC)	WES	[69]
NINL (Ninein like)	20p11.21	*NINL* was identified as a high confidence risk gene.	13 GTS multiplex families (Caucasian)	WES, genotyping	[44]

Gene	Chromosome	Finding	Sample Size (Ancestry)	Analysis	Reference
SYNDIG1 (Synapse differentiation inducing 1)	20p11.21	Identification of *de novo* mutation.	9 GTS trios; 1 quartet with GTS CC MZ twins (NA)	WES	[96]
POFUT1 (Protein O-fucosyltransferase 1)	20q11.21	*POFUT1* was identified as a high confidence risk gene.	13 GTS multiplex families (Caucasian)	WES, genotyping	[44]
AHCY (Adenosylhomocysteinase)	20q11.22	*AHCY* was identified as a low confidence risk gene.	13 GTS multiplex families (Caucasian)	WES, genotyping	[44]
DDX27 (DEAD-box helicase 27)	20q13.13	Identification of *de novo* mutation.	9 GTS trios; 1 quartet with GTS CC MZ twins (NA)	WES	[96]
HRH3 (Histamine receptor H3)	20q13.33	No association between GTS and *HRH3*.	465 GTS/CTD probands in 412 families (USA, Europe, South Korea); Replication: 1,285 GTS; Replication: 4,964 controls (European, Ashkenazi Jewish and French Canadian)	Association (TDT)	[73]
RTEL1 (Regulator of telomere elongation helicase 1)	20q13.33	Identification of *de novo* mutation.	9 GTS trios; 1 quartet with GTS CC MZ twins (NA)	WES	[96]
URB1 (URB1 ribosome biogenesis homolog)	21q22.11	*De novo* missense mutation identified in *URB1* in GTS individuals.	119 GTS+ASD children; 2,603 ASD children (SSC)	GSEA	[76]
PCNT (Pericentrin)	21q22.3	Association between GTS and SNPs rs17371795, rs2839227, and rs2839228. rs2839227 remained significant after Bonferroni correction.	407 GTS trios; 506 controls (Han Chinese)	Association (TDT, HRR)	[117]
KB-67B5.12 (PIK3CA pseudogene)	22q11.1	Identification of *de novo* mutation.	9 GTS trios; 1 quartet with GTS CC MZ twins (NA)	WES	[96]
ADORA2A (Adenosine A2a receptor)	22q11.23	Association between GTS and SNP rs5751876.	162 GTS; 270 controls (Polish)	Association	[104]
ACO2 (Aconitase 2)	22q13.2	Association between GTS and rs5758265 {ha}.	232,964 GTS/AN/ADHD/ASD/BP/ MD/OCD/SCZ (incl. 4,645 GTS (European, North American)); 494,162 controls (Mixed)	GWAS	[80]
ADSL (Adenylosuccinate lyase)	22q13.2	Association between GTS and rs5758265 {ha}.	232,964 GTS/AN/ADHD/ASD/BP/	GWAS	[80]

Gene	Chromosome	Finding	Sample Size (Ancestry)	Analysis	Reference
			MD/OCD/SCZ (incl. 4,645 GTS (European, North American)); 494,162 controls (Mixed)		
CACNG2 (Calcium voltage-gated channel auxiliary subunit gamma 2)	22q12.3	No association found between GTS and *CACNG2.*	201 GTS (from families and singletons); 253 controls (USA); replication: 44 GTS (from families and singletons); 73 controls (Spain)	Genotyping, association (TDT)	[27]
CHADL (Chondroadherin like)	22q13.2	Association between GTS and rs5758265 {g, ha, hf}.	232,964 GTS/AN/ADHD/ASD/BP/ MD/OCD/SCZ (incl. 4,645 GTS (European, North American)); 494,162 controls (Mixed)	GWAS	[80]
DESI1 (Desumoylating isopeptidase 1)	22q13.2	Association between GTS and rs5758265 {ha}.	232,964 GTS/AN/ADHD/ASD/BP/ MD/OCD/SCZ (incl. 4,645 GTS (European, North American)); 494,162 controls (Mixed)	GWAS	[80]
EP300 (E1A binding protein P300)	22q13.2	Association between GTS and rs5758265 {fg, ha, hf}.	232,964 GTS/AN/ADHD/ASD/BP/ MD/OCD/SCZ (incl. 4,645 GTS (European, North American)); 494,162 controls (Mixed)	GWAS	[80]
L3MBTL2 (L3MBTL histone methyl-lysine binding protein 2)	22q13.2	Association between GTS and rs5758265 {g, ha}.	232,964 GTS/AN/ADHD/ASD/BP/ MD/OCD/SCZ (incl. 4,645 GTS (European, North American)); 494,162 controls (Mixed)	GWAS	[80]
PMM1 (Phosphomannomutase 1)	22q13.2	Association between GTS and rs5758265 {ha}.	232,964 GTS/AN/ADHD/ASD/BP/ MD/OCD/SCZ (incl. 4,645 GTS (European, North American)); 494,162 controls (Mixed)	GWAS	[80]
RANGAP1 (Ran GTPase activating protein 1)	22q13.2	Association between GTS and rs5758265 {ha, hf, tss}.	232,964 GTS/AN/ADHD/ASD/BP/	GWAS	[80]

Gene	Chromosome	Finding	Sample Size (Ancestry)	Analysis	Reference
			MD/OCD/SCZ (incl. 4,645 GTS (European, North American)); 494,162 controls (Mixed)		
RBX1 (Ring-box 1)	22q13.2	Association between GTS and rs5758265 {ha, hf}.	232,964 GTS/AN/ADHD/ASD/BP/ MD/OCD/SCZ (incl. 4,645 GTS (European, North American)); 494,162 controls (Mixed)	GWAS	[80]
SLC25A17 (Solute carrier family 25 member 17)	22q13.2	Association between GTS and rs5758265 {tss}.	232,964 GTS/AN/ADHD/ASD/BP/ MD/OCD/SCZ (incl. 4,645 GTS (European, North American)); 494,162 controls (Mixed)	GWAS	[80]
ST13 (ST13 Hsp70 interacting protein)	22q13.2	Association between GTS and rs5758265 {fg}.	232,964 GTS/AN/ADHD/ASD/BP/ MD/OCD/SCZ (incl. 4,645 GTS (European, North American)); 494,162 controls (Mixed)	GWAS	[80]
TEF (TEF transcription factor, PAR BZIP family member)	22q13.2	Association between GTS and rs5758265 {ha}.	232,964 GTS/AN/ADHD/ASD/BP/ MD/OCD/SCZ (incl. 4,645 GTS (European, North American)); 494,162 controls (Mixed)	GWAS	[80]
TOB2 (Transducer of ERBB2, 2)	22q13.2	Association between GTS and rs5758265 {ha, hf}.	232,964 GTS/AN/ADHD/ASD/BP/ MD/OCD/SCZ (incl. 4,645 GTS (European, North American)); 494,162 controls (Mixed)	GWAS	[80]
XPNPEP3 (X-prolyl aminopeptidase 3)	22q13.2	Association between GTS and rs5758265 {fg}.	232,964 GTS/AN/ADHD/ASD/BP/ MD/OCD/SCZ (incl. 4,645 GTS (European, North American)); 494,162 controls (Mixed)	GWAS	[80]
XRCC6 (X-ray repair cross complementing 6)	22q13.2	Association between GTS and rs5758265 {ha}.	232,964 GTS/AN/ADHD/ASD/BP/	GWAS	[80]

Gene	Chromosome	Finding	Sample Size (Ancestry)	Analysis	Reference
			MD/OCD/SCZ (incl. 4,645 GTS (European, North American)); 494,162 controls (Mixed)		
ZC3H7B (Zinc finger CCCH-type containing 7B)	22q13.2	Association between GTS and rs5758265 {ha}.	232,964 TS/AN/ADHD/ASD/BP/ MD/OCD/SCZ (incl. 4,645 GTS (European, North American)); 494,162 controls (Mixed)	GWAS	[80]
NLGN4 (Neuroligin 4)	Xp22.33	Association between GTS, ASD as well as other disorders and a deletion of exons 4, 5, and 6.	1 family with GTS, ASD, ADHD, *i.a.* (English, Irish)	Sequence analysis	[118]
MAOB (Monoamine oxidase B)	Xp11.3	No association found between GTS and *MAOB.*	201 GTS (from families and singletons); 253 controls (USA); replication: 44 GTS (from families and singletons); 73 controls (Spain)	Genotyping, association (TDT)	[27]
NLGN3 (Neuroligin 3)	Xq13.1	*NLGN3* was identified as a high confidence risk gene.	13 GTS multiplex families (Caucasian)	WES, genotyping	[44]
PABPC5 (Poly(A) binding protein cytoplasmic 5)	Xq21.31	Two microduplications (15q13.3 and Xq21.31) present in family; *PABPC5* were within the Xq duplication.	One GTS family (NA)	Breakpoint mapping	[99]
PCDH11X (Protocadherin 11 X-linked)	Xq21.31-.32	Two microduplications (15q13.3 and Xq21.31) present in family; *PCDH11X* were within the Xq duplication.	One GTS family (NA)	Breakpoint mapping	[99]
CAPN6 (Calpain 6)	Xq23	*CAPN6* was identified as a low confidence risk gene.	13 GTS multiplex families (Caucasian)	WES, genotyping	[44]
GRIA3 (Glutamate ionotropic receptor AMPA type subunit 3)	Xq25	No association found between GTS and *GRIA3.*	201 GTS (from families and singletons); 253 controls (USA); replication: 44 GTS (from families and singletons); 73 controls (Spain)	Genotyping, association (TDT)	[27]
No linkage found between GTS and any markers.			Seven multigenerational GTS families (North American)	Genome-wide linkage analysis	[119]
No SNP signals reached genome-wide threshold of significance.			1,285 GTS; 4,964 controls (European, Ashkenazi Jewish, and French Canadian);	GWAS	[111]

Gene	Chromosome	Finding	Sample Size (Ancestry)	Analysis	Reference
			Replication: 211 GTS; 285 controls (Latin American)		
		No SNP signals reached genome-wide threshold of significance.	217 GTS trios (French Canadian)	Family-based GWAS, TDT	[120]
		No SNP signals reached genome-wide threshold of significance.	2,723 cases (1,310 OCD, 834 GTS, 579 OCD+GTS/CTD); 290 OCD trios; 5,667 controls (Mixed)	GWAS	[121]
		No signals reached epigenome-wide threshold of significance.	1,057 families with 188 indv. with tic disorders + 1,490 controls (Dutch)	EWAS	[122]

SNP: Single nucleotide polymorphism; VNTR: Variable number tandem repeat; LGD: Likely gene disrupting; Indv.: Individuals; MZ: Monozygotic; DC: Discordant; CC: Concordant; GTS: Gilles de la Tourette syndrome; CTD: Chronic tic disorder; CMT: Chronic multiple tics; ASD: Autism spectrum disorder; TD: Tic disorder; OCD: Obsessive-compulsive disorder; ADHD: Attention-deficit hyperactivity disorder; AN: Anorexia nervosa; BP: Bipolar disorder; MD; Major depression; SCZ: Schizophrenia; SMS: Smith-Magenis syndrome; SSC: Simons Simplex Collection; NA: Ancestry information not available; CNV: Copy number variation; TDT: Transmission disequilibrium test; MDS: Multidimensional scaling analysis; HRR: Haplotype relative risk; WES: Whole exome sequencing; GSEA: Gene set enrichment analysis; GWAS: Genome-wide association study; EWAS: Epigenome-wide association study.

From [80]: {g}: gene containing index SNP; {fg}: credible SNP gene; {q}: brain cis-eQTLs; {h}: hi-C interacting gene based on FUMA; {hf}: hi-C-based interaction between associated SNP and target gene in the fetal brain; {ha}: hi-C-based interaction in the adult brain; {tss}: transcription start sites.

Supplementary Table S1B

Region	Genes in region previously implicated in GTS	Finding	Sample Size (Ancestry)	Analysis	Reference
1p36.33		Association between GTS and miR-429 expression. miR-429 locates to 1p36.33.	6 TS; 3 controls; replication: 52 GTS; 15 controls (Caucasian)	Transcriptome analysis	[123]
1p31.1	*NEGR1, LHX8*	Association between GTS/tics and locus D1S207.	One 260 indv. pedigree incl. 108 GTS/Tic indv. (Utah)	Genome-wide linkage analysis	[124]
2p23.3		Association between GTS and locus D2S144.	238 nuclear families with GTS/CTD indv. (NA)	Whole genome scan, linkage analysis	[125]
2p12		Association between GTS and locus D2S139.	91 nuclear families with 107 GTS indv. (Afrikaner)	Association (TDT, HRR)	[126]
2p11.2	*MRPL35*	Association between GTS and loci D2S440 and D2S1790.	40 GTS; 60 GTS; 96 controls (Afrikaner)	Whole genome scan, association	[127]
2q24.1	Nearest gene: *NR4A2*	Association between GTS and rs1226412.	232,964 GTS/AN/ADHD/ASD/BP/ MD/OCD/SCZ (incl. 4,645 GTS (European, North American)); 494,162 controls (Mixed)	GWAS	[80]
3p21-14	*STAB1, CELSR2*	Association between GTS/CTD and polymorphisms surrounding locus D3S11.	One 122 indv. family incl. 46 GTS/CTD (NA)	Linkage analysis	[128]
3p14.3		Association between GTS/tics and locus D3S1289.	One 260 indv. pedigree incl. 108 GTS/Tic indv. (Utah)	Genome-wide linkage analysis	[124]
3q29	*OPA1*	Association between GTS and locus D3S1311.	One GTS multiplex pedigree with 27 members (Dutch)	Linkage analysis	[129]
4q31.21		Suggested association between GTS and loci D4S1644 and D4S1625.	76 families with 110 GTS sib-pairs (NA)	Whole genome scan	[130]
4q34-35		Association between GTS and the 4q34-35 region.	77 GTS sib pairs (NA)	Whole genome scan, linkage analysis	[131]
5p13.3-5q12.1	*HCN1, FGF10, RICTOR, SLC1A3, GDNF*	No association found between GTS and genes in the D5S1506-D5S76 region.	1 large GTS family (Canadian); 241 GTS children in 171 nuclear families (NA)	Linkage, Association (TDT)	[79]
5q21.2		Association between GTS and rs12658451.	232,964 GTS/AN/ADHD/ASD/BP/	GWAS	[80]

			MD/OCD/SCZ (incl. 4,645 GTS (European, North American)); 494,162 controls (Mixed)		
5q34	*WWC1*	No major association found between GTS/CMT and the D5S2050-D5S400 region.	One 85 indv. pedigree incl. 49 GTS/CMT indv. (British)	Linkage analysis	[132]
5q35.2-35.3	*DRD1*	Association between GTS and the 5q35.2-35.3 region.	77 GTS sib pairs (NA)	Whole genome scan, linkage analysis	[131]
6p25.1		Association between GTS and locus D6S477.	40 GTS; 60 GTS; 96 controls (Afrikaner)	Whole genome scan, association	[127]
7q31	*IMMPL2*	Association between GTS and loci D7S522 and D7S523, and a tendency for association between GTS and locus D7S1516.	86 GTS trios (French Canadian)	Association (TDT)	[133]
8p21.3-22		Suggested association between GTS and loci D8S1106 and D8S136.	76 families with 110 GTS sib-pairs (NA)	Whole genome scan	[130]
8q22.1		Association between GTS and marker GATA28F12.	91 nuclear families with 107 GTS indv. (Afrikaner)	Association (TDT, HRR)	[126]
8q22.2-23.1		Association between GTS and loci D8S257 and D8S1132.	40 GTS; 60 GTS; 96 controls (Afrikaner)	Whole genome scan, association	[127]
10p15.1-14		No major association found between GTS/CMT and the D10S591-D10S189 region.	One 85 indv. pedigree incl. 49 GTS/CMT indv. (British)	Linkage analysis	[132]
11p15.3		Nominally significant association between GTS and rs11603305.	465 GTS/CTD probands in 412 families (USA, Europe, South Korea); Replication: 1,285 GTS; Replication: 4,964 controls (European, Ashkenazi Jewish and French Canadian)	Association (TDT)	[73]
11q24	*ROBO3, ROBO4*	No association found between GTS and 11q24.	199 nuclear GTS families (French Canadian)	Association (TDT)	[134]
11q24.1		Association between GTS and locus D11S1377.	91 nuclear families with 107 GTS indv. (Afrikaner)	Association (TDT, HRR)	[126]
11q24.1-24.2	*ROBO3, ROBO4*	Association between GTS and loci D11S1377 and D11S933.	40 GTS; 60 GTS; 96 controls (Afrikaner)	Whole genome scan, association	[127]

			One 127 indv. family incl. 20 GTS indv. and 20 indv. with tics (French Canadian)	Linkage analysis	[135]
12q23.1 (previously 12q22)	Near *NTN4*	Association between GTS and rs2060546.	609 GTS; 610 controls (European/French Canadian)	Association, GWAS-meta-analysis	[136]
		Association between GTS and rs2060546 in the meta-analysis, not in the Danish cohort alone.	240 GTS; 1,006 controls (Danish); Meta-analysis: 1,556 GTS; 6,029 controls (mixed)	Association, GWAS-meta-analysis	[137]
13q12.3-13.1	*KATNAL1*	No major association found between GTS/CMT and the D13S217-D13S171 region.	One 85 indv. pedigree incl. 49 GTS/CMT indv. (British)	Linkage analysis	[132]
14q11.2		Association between GTS and locus D14S1003.	40 GTS; 60 GTS; 96 controls (Afrikaner)	Whole genome scan, association	[127]
14q31.1		Suggestive association between GTS and 14q31.1.	One 30 indv. multiplex GTS/CMT/OCD family (Italian)	Linkage analysis	[138]
16p13.11		No significant association between GTS and 16p13.11.	1,086 GTS; 1,613 OCD; 1,789 controls (mostly European ancestry)	Genome-wide CNV analysis, Cross-disorder study	[139]
17q11.2	*SLC6A4*	Identification of microdeletion of chromosome 17q11.2 in case indv.	One GTS+SMS indv.	Chromosome analysis, case report	[140]
17q25		Association between GTS and the 17q25 region.	77 GTS sib pairs (NA)	Whole genome scan, linkage analysis	[131]
17q25	*TBCD*	Association between GTS and chromosome 17q25.	4 multiplex pedigrees (462 indv. incl. 105 GTS) (North American); 96 nuclear families (330 indv. incl. 151 GTS) (mostly European ancestry)	Linkage analysis, association	[141]
20q13.2		Association between GTS and loci D20S1085 and D20S468.	40 GTS; 60 GTS; 96 controls (Afrikaner)	Whole genome scan, association	[127]
		No association found between GTS and loci D20S1085 and D20S468.	91 nuclear families with 107 GTS indv. (Afrikaner)	Association (TDT, HRR)	[126]
21q22.11-22.13	*URB1*	Association between GTS and marker GATA45C03 and locus D21S1252.	40 GTS; 60 GTS; 96 controls (Afrikaner)	Whole genome scan, association	[127]
		No association found between GTS and marker GATA45C03 and locus D21S1252.	91 nuclear families with 107 GTS indv. (Afrikaner)	Association (TDT, HRR)	[126]

22q11.2	*COMT*	Suggested association between GTS and chromosome 22q11.2 deletion.	One indv. with GTS and chr22q11.2 deletion syndrome	Chromosome analysis, case report	[142]
Xq28	*VAMP7, SPRY3*	Identification of a duplication at Xq28.	One indv. with GTS *i.a.* (Caucasian)	Chromosome analysis, case report	[143]

Indv.: Individuals; GTS: Gilles de la Tourette syndrome; CTD: Chronic tic disorder; CMT: Chronic multiple tics; ASD: Autism spectrum disorder; OCD: Obsessive-compulsive disorder; ADHD: Attention-deficit hyperactivity disorder; AN: Anorexia nervosa; BP: Bipolar disorder; MD; Major depression; SCZ: Schizophrenia; SMS: Smith-Magenis syndrome; NA: Ancestry information not available; CNV: Copy number variation; TDT: Transmission disequilibrium test; HRR: Haplotype relative risk; GWAS: Genome-wide association study.

Supplementary Table S1C

Region	Genes on list in region	Finding	Sample Size (Ancestry)	Method	Reference
dup(7)(q22.1-q31.1)	*CLDN15, IMMP2L*	Case report of a GTS individual with a *de novo* duplication.	1 GTS indv (NA)	Chromosome analysis, case report	[144]
t(6,8)		Description of two GTS families with similar balanced translocation.	Two families with GTS individuals (NA)	Breakpoint mapping	[145]
inv(18)(q21.1-q22.2)	*DCC*	Description of a CTD+OCD individual with epigenetic abnormalities in the region of the inversion.	One CTD+OCD indv (Korean)	Breakpoint mapping	[146]
inv(15)(q13;q22.3)		Description of an individual with chorea and tics with a de novo inversion.	One indv. with chorea and tics (Caucasian)	Breakpoint mapping, sequence analysis, case report	[147]
t(6;17)(q21;p11)		Description of a translocation found in father and son, both with GTS.	Father and son with GTS (NA)	Chromosome analysis, case report	[148]
t(6,22)(q16.2;p13)		Identification of chromosomal rearrangements in the GTS+OCD individual.	One GTS+OCD indv. (NA)	Chromosome analysis, WGS, case report	[149]
t(3;9)(q25;q34)	*AADAC, DBH, OLFM1*	Identification of translocation in one GTS individual.	205 GTS indv. (Danish)	Chromosome analysis	[150]
t(3;5)(q25;q31)	*AADAC, MATR3*	Identification of translocation in one GTS individual.	205 GTS indv. (Danish)	Chromosome analysis	[150]

Dup: duplication; t: translocation; inv: inversion; Indv.: Individuals; GTS: Gilles de la Tourette syndrome; CTD: Chronic tic disorder; OCD: Obsessive-compulsive disorder; NA: Ancestry information not available.

References

[1] Abelson, J. F.; Kwan, K. Y.; O'Roak, B. J.; Baek, D. Y.; Stillman, A. A.; Morgan, T. M.; Mathews, C. A.; Pauls, D. L.; Rasin, M.-R.; Gunel, M.; et al. Sequence Variants in SLITRK1 Are Associated with Tourette's Syndrome. *Science.*, **2005**. https://doi.org/10.1126/science.1116502.

[2] Miranda, D. M.; Wigg, K.; Kabia, E. M.; Feng, Y.; Sandor, P.; Barr, C. L. Association of SLITRK1 to Gilles de La Tourette Syndrome. *Am. J. Med. Genet. Part B Neuropsychiatr. Genet.*, **2009**. https://doi.org/10.1002/ajmg.b.30840.

[3] Karagiannidis, I.; Rizzo, R.; Tarnok, Z.; Wolanczyk, T.; Hebebrand, J.; Nöthen, M. M.; Lehmkuhl, G.; Farkas, L.; Nagy, P.; Barta, C.; et al. Replication of Association between a SLITRK1 Haplotype and Tourette Syndrome in a Large Sample of Families. *Mol. Psychiatry*, **2012**. https://doi.org/10.1038/mp.2011.151.

[4] Inai, A.; Tochigi, M.; Kuwabara, H.; Nishimura, F.; Kato, K.; Eriguchi, Y.; Shimada, T.; Furukawa, M.; Kawamura, Y.; Sasaki, T.; et al. Analysis of SLITRK1 in Japanese Patients with Tourette Syndrome Using a Next-Generation Sequencer. *Psychiatr. Genet.*, **2015**. https://doi.org/10.1097/YPG.0000000000000104.

[5] O'Roak, B. J.; Morgan, T. M.; Fishman, D. O.; Saus, E.; Alonso, P.; Gratacòs, M.; Estivill, X.; Teltsh, O.; Kohn, Y.; Kidd, K. K.; et al. Additional Support for the Association of SLITRK1 Var321 and Tourette Syndrome. *Mol. Psychiatry*, **2010**. https://doi.org/10.1038/mp.2009.105.

[6] Alexander, J.; Potamianou, H.; Xing, J.; Deng, L.; Karagiannidis, I.; Tsetsos, F.; Drineas, P.; Tarnok, Z.; Rizzo, R.; Wolanczyk, T.; et al. Targeted Re-Sequencing Approach of Candidate Genes Implicates Rare Potentially Functional Variants in Tourette Syndrome Etiology. *Front. Neurosci.*, **2016**. https://doi.org/10.3389/fnins.2016.00428.

[7] Depienne, C.; Ciura, S.; Trouillard, O.; Bouteiller, D.; Leitão, E.; Nava, C.; Keren, B.; Marie, Y.; Guegan, J.; Forlani, S.; et al. Association of Rare Genetic Variants in Opioid Receptors with Tourette Syndrome. *Tremor Other Hyperkinet. Mov. (N. Y).*, **2019**. https://doi.org/10.7916/tohm.v0.693.

[8] Chou, I. C.; Wan, L.; Liu, S. C.; Tsai, C. H.; Tsai, F. J. Association of the Slit and Trk-like 1 Gene in Taiwanese Patients With Tourette Syndrome. *Pediatr. Neurol.*, **2007**. https://doi.org/10.1016/j.pediatrneurol.2007.06.017.

[9] Zimprich, A.; Hatala, K.; Riederer, F.; Stogmann, E.; Aschauer, H. N.; Stamenkovic, M. Sequence Analysis of the Complete SLITRK1 Gene in Austrian Patients with Tourette's Disorder. *Psychiatr. Genet.*, **2008**. https://doi.org/10.1097/YPG.0b013e3283060f6f.

[10] Fabbrini, G.; Pasquini, M.; Aurilia, C.; Berardelli, I.; Breedveld, G.; Oostra, B. A.; Bonifati, V.; Berardelli, A. A Large Italian Family with Gilles de La Tourette Syndrome: Clinical Study and Analysis of the SLITRK1 Gene. *Mov. Disord.*, **2007**. https://doi.org/10.1002/mds.21697.

[11] Deng, H.; Le, W. D.; Xie, W. J.; Jankovic, J. Examination of the SLITRK1 Gene in Caucasian Patients with Tourette Syndrome. *Acta Neurol. Scand.*, **2006**. https://doi.org/10.1111/j.1600-0404.2006.00706.x.

[12] Scharf, J. M.; Moorjani, P.; Fagerness, J.; Platko, J. V.; Illmann, C.; Galloway, B.; Jenike, E.; Stewart, S. E.; Pauls, D. L.; Cath, D.; et al. Lack of Association between SLITRK1 Var321 and Tourette Syndrome in a Large Family-Based Sample. *Neurology*, **2008**. https://doi.org/10.1212/01.wnl.0000296833.25484.bb.

[13] Keen-Kim, D.; Mathews, C. A.; Reus, V. I.; Lowe, T. L.; Herrera, L. D.; Budman, C. L.; Gross-Tsur, V.; Pulver, A. E.; Bruun, R. D.; Erenberg, G.; et al. Overrepresentation of Rare Variants in a Specific Ethnic Group May Confuse Interpretation of Association Analyses. *Hum. Mol. Genet.*, **2006**.

https://doi.org/10.1093/hmg/ddl408.

[14] Yasmeen, S.; Melchior, L.; Bertelsen, B.; Skov, L.; Debes, N. M.; Tümer, Z. Sequence Analysis of SLITRK1 for Var321 in Danish Patients with Tourette Syndrome and Review of the Literature. *Psychiatr. Genet.*, **2013**. https://doi.org/10.1097/YPG.0b013e328360c880.

[15] Herzberg, I.; Valencia-Duarte, A. V.; Kay, V. A.; White, D. J.; Müller, H.; Rivas, I. C.; Mesa, S. C.; Cuartas, M.; García, J.; Bedoya, G.; et al. Association of DRD2 Variants and Gilles de La Tourette Syndrome in a Family-Based Sample from a South American Population Isolate. *Psychiatr. Genet.*, **2010**. https://doi.org/10.1097/YPG.0b013e32833a215a.

[16] Müller-Vahl, K. R.; Loeber, G.; Kotsiari, A.; Müller-Engling, L.; Frieling, H. Gilles de La Tourette Syndrome Is Associated with Hypermethylation of the Dopamine D2 Receptor Gene. *J. Psychiatr. Res.*, **2017**. https://doi.org/10.1016/j.jpsychires.2016.11.004.

[17] Lee, C. C.; Chou, I. C.; Tsai, C. H.; Wang, T. R.; Li, T. C.; Tsai, F. J. Dopamine Receptor D2 Gene Polymorphisms Are Associated in Taiwanese Children with Tourette Syndrome. *Pediatr. Neurol.*, **2005**. https://doi.org/10.1016/j.pediatrneurol.2005.05.005.

[18] Comings, D. E.; Comings, B. G.; Muhleman, D.; Dietz, G.; Shahbahrami, B.; Tast, D.; Knell, E.; Kocsis, P.; Baumgarten, R.; Kovacs, B. W.; et al. The Dopamine D2 Receptor Locus as a Modifying Gene in Neuropsychiatric Disorders. *JAMA J. Am. Med. Assoc.*, **1991**. https://doi.org/10.1001/jama.1991.03470130073032.

[19] Comings, D. E.; Wu, S.; Chiu, C.; Ring, R. H.; Gade, R.; Ahn, C.; MacMurray, J. P.; Dietz, G.; Muhleman, D. Polygenic Inheritance of Tourette Syndrome, Stuttering, Attention Deficit Hyperactivity, Conduct, and Oppositional Defiant Disorder: The Additive and Subtractive Effect of the Three Dopaminergic Genes - DRD2, DβH, and DAT1. *Am. J. Med. Genet. - Semin. Med. Genet.*, **1996**. https://doi.org/10.1002/(SICI)1096-8628(19960531)67:3<264::AID-AJMG4>3.0.CO;2-N.

[20] Yuan, A.; Su, L.; Yu, S.; Li, C.; Yu, T.; Sun, J. Association between DRD2/ANKK1 TaqIA Polymorphism and Susceptibility with Tourette Syndrome: A Meta-Analysis. *PLoS One*, **2015**. https://doi.org/10.1371/journal.pone.0131060.

[21] Nothen, M. M.; Hebebrand, J.; Knapp, M.; Hebebrand, K.; Camps, A.; Von Gontard, A.; Wettke-Schafer, R.; Lisch, S.; Cichon, S.; Poustka, F.; et al. Association Analysis of the Dopamine D2 Receptor Gene in Tourette's Syndrome Using the Haplotype Relative Risk Method. *Am. J. Med. Genet.*, **1994**. https://doi.org/10.1002/ajmg.1320540311.

[22] Díaz-Anzaldúa, A.; Joober, R.; Rivière, J. B.; Dion, Y.; Lespérance, P.; Richer, F.; Chouinard, S.; Rouleau, G. A. Tourette Syndrome and Dopaminergic Genes: A Family-Based Association Study in the French Canadian Founder Population. *Mol. Psychiatry*, **2004**. https://doi.org/10.1038/sj.mp.4001411.

[23] Gelernter, J.; Pauls, D. L.; Leckman, J.; Kidd, K. K.; Kurlan, R. D2 Dopamine Receptor Alleles Do Not Influence Severity of Tourette's Syndrome: Results from Four Large Kindreds. *Arch. Neurol.*, **1994**. https://doi.org/10.1001/archneur.1994.00540160099012.

[24] Gelernter, J.; Pakstis, A. J.; Pauls, D. L.; Kurlan, R.; Gancher, S. T.; Civelli, O.; Grandy, D.; Kidd, K. K. Gilles de La Tourette Syndrome Is Not Linked to D2-Dopamine Receptor. *Arch. Gen. Psychiatry*, **1990**. https://doi.org/10.1001/archpsyc.1990.01810230089014.

[25] Devor, E. J.; Grandy, D. K.; Civelli, O.; Litl, M.; Burgess, A. K.; Isenberg, K. E.; Van De Wetering, B. J. M.; Oostra, B. Genetic Linkage Is Excluded for the D2-Dopamine Receptor ΛHD2G1 and Flanking Loci on Chromosome 11q22-Q23 in Tourette Syndrome. *Hum. Hered.*, **1990**. https://doi.org/10.1159/000153914.

[26] Brett, P. M.; Curtis, D.; Robertson, M. M.; Gurling, H. M. D. The Genetic Susceptibility to Gilles de La Tourette Syndrome in a Large Multiple Affected British

Kindred: Linkage Analysis Excludes a Role for the Genes Coding for Dopamine D1, D2, D3, D4, D5 Receptors, Dopamine Beta Hydroxylase, Tyrosinase, and Tyrosine. *Biol. Psychiatry*, **1995**. https://doi.org/10.1016/0006-3223(94)00161-U.

[27] Huertas-Fernández, I.; Gómez-Garre, P.; Madruga-Garrido, M.; Bernal-Bernal, I.; Bonilla-Toribio, M.; Martín-Rodríguez, J. F.; Cáceres-Redondo, M. T.; Vargas-González, L.; Carrillo, F.; Pascual, A.; et al. GDNF Gene Is Associated with Tourette Syndrome in a Family Study. *Mov. Disord.*, **2015**. https://doi.org/10.1002/mds.26279.

[28] Grice, D. E.; Leckman, J. F.; Pauls, D. L.; Kurlan, R.; Kidd, K. K.; Pakstis, A. J.; Chang, F. M.; Buxbaum, J. D.; Cohen, D. J.; Gelernter, J. Linkage Disequilibrium between an Allele at the Dopamine D4 Receptor Locus and Tourette Syndrome, by the Transmission-Disequilibrium Test. *Am. J. Hum. Genet.*, **1996**, *59*, 644–652.

[29] Liu, S.; Cui, J.; Zhang, X.; Wu, W.; Niu, H.; Ma, X.; Xu, H.; Yi, M. Variable Number Tandem Repeats in Dopamine Receptor D4 in Tourette's Syndrome. *Mov. Disord.*, **2014**. https://doi.org/10.1002/mds.26027.

[30] Cruz, C.; Camarena, B.; King, N.; Páez, F.; Sidenberg, D.; De La Fuente, J. R.; Nicolini, H. Increased Prevalence of the Seven-Repeat Variant of the Dopamine D4 Receptor Gene in Patients with Obsessive-Compulsive Disorder with Tics. *Neurosci. Lett.*, **1997**. https://doi.org/10.1016/S0304-3940(97)00523-5.

[31] Hebebrand, J.; Nöthen, M. M.; Ziegler, A.; Klug, B.; Neidt, H.; Eggermann, K.; Lehmkuhl, G.; Poustka, F.; Schmidt, M. H.; Propping, P.; et al. Nonreplication of Linkage Disequilibrium between the Dopamine D4 Receptor Locus and Tourette Syndrome. *Am. J. Hum. Genet.*, **1997**. https://doi.org/10.1016/s0002-9297(07)64298-0.

[32] Comings, D. E.; Gonzalez, N.; Wu, S.; Gade, R.; Muhleman, D.; Saucier, G.; Johnson, P.; Verde, R.; Rosenthal, R. J.; Lesieur, H. R.; et al. Studies of the 48 Bp Repeat Polymorphism of the DRD4 Gene in Impulsive, Compulsive, Addictive Behaviors: Tourette Syndrome, ADHD, Pathological Gambling, and Substance Abuse. *Am. J. Med. Genet. - Neuropsychiatr. Genet.*, **1999**. https://doi.org/10.1002/(SICI)1096-8628(19990820)88:4<358::AID-AJMG13>3.0.CO;2-G.

[33] Tarnok, Z.; Ronai, Z.; Gervai, J.; Kereszturi, E.; Gadoros, J.; Sasvari-Szekely, M.; Nemoda, Z. Dopaminergic Candidate Genes in Tourette Syndrome: Association between Tic Severity and 3' UTR Polymorphism of the Dopamine Transporter Gene. *Am. J. Med. Genet. Part B Neuropsychiatr. Genet.*, **2007**. https://doi.org/10.1002/ajmg.b.30517.

[34] Yoon, D. Y.; Rippel, C. A.; Kobets, A. J.; Morris, C. M.; Lee, J. E.; Williams, P. N.; Bridges, D. D.; Vandenbergh, D. J.; Shugart, Y. Y.; Singer, H. S. Dopaminergic Polymorphisms in Tourette Syndrome: Association with the DAT Gene (SLC6A3). *Am. J. Med. Genet. Part B Neuropsychiatr. Genet.*, **2007**. https://doi.org/10.1002/ajmg.b.30466.

[35] Barr, C. L.; Wigg, K. G.; Zovko, E.; Sandor, P.; Tsui, L. C. No Evidence for a Major Gene Effect of the Dopamine D4 Receptor Gene in the Susceptibility to Gilles de La Tourette Syndrome in Five Canadian Families. *Am. J. Med. Genet. - Semin. Med. Genet.*, **1996**. https://doi.org/10.1002/(SICI)1096-8628(19960531)67:3<301::AID-AJMG6>3.0.CO;2-P.

[36] Rowe, D. C.; Stever, C.; Gard, J. M. C.; Cleveland, H. H.; Sanders, M. L.; Abramowitz, A.; Kozol, S. T.; Mohr, J. H.; Sherman, S. L.; Waldman, I. D. The Relation of the Dopamine Transporter Gene (DAT1) to Symptoms of Internalizing Disorders in Children. *Behav. Genet.*, **1998**. https://doi.org/10.1023/A:1021427314941.

[37] Ercan-Sencicek, A. G.; Stillman, A. A.; Ghosh, A. K.; Bilguvar, K.; O'Roak, B. J.; Mason, C. E.; Abbott, T.; Gupta, A.; King, R. A.; Pauls, D. L.; et al. L-Histidine Decarboxylase and Tourette's Syndrome. *N. Engl. J. Med.*, **2010**. https://doi.org/10.1056/nejmoa0907006.

[38] Karagiannidis, I.; Dehning, S.; Sandor, P.; Tarnok, Z.; Rizzo, R.; Wolanczyk, T.; Madruga-Garrido, M.; Hebebrand, J.; Nöthen, M. M.; Lehmkuhl, G.; et al. Support of the Histaminergic Hypothesis in Tourette Syndrome: Association of the Histamine Decarboxylase Gene in a Large Sample of Families. *J. Med. Genet.*, **2013**. https://doi.org/10.1136/jmedgenet-2013-101637.

[39] Castellan Baldan, L.; Williams, K. A.; Gallezot, J. D.; Pogorelov, V.; Rapanelli, M.; Crowley, M.; Anderson, G. M.; Loring, E.; Gorczyca, R.; Billingslea, E.; et al. Histidine Decarboxylase Deficiency Causes Tourette Syndrome: Parallel Findings in Humans and Mice. *Neuron*, **2014**. https://doi.org/10.1016/j.neuron.2013.10.052.

[40] Lei, J.; Deng, X.; Zhang, J.; Su, L.; Xu, H.; Liang, H.; Huang, X.; Song, Z.; Deng, H. Mutation Screening of the HDC Gene in Chinese Han Patients with Tourette Syndrome. *Am. J. Med. Genet. Part B Neuropsychiatr. Genet.*, **2012**. https://doi.org/10.1002/ajmg.b.32003.

[41] Dong, H.; Liu, W.; Liu, M.; Xu, L.; Li, Q.; Zhang, R.; Zhang, X.; Liu, S. Investigation of a Possible Role for the Histidine Decarboxylase Gene in Tourette Syndrome in the Chinese Han Population: A Family-Based Study. *PLoS One*, **2016**. https://doi.org/10.1371/journal.pone.0160265.

[42] Moya, P. R.; Wendland, J. R.; Rubenstein, L. M.; Timpano, K. R.; Heiman, G. A.; Tischfield, J. A.; King, R. A.; Andrews, A. M.; Ramamoorthy, S.; Mcmahon, F. J.; et al. Common and Rare Alleles of the Serotonin Transporter Gene, SLC6A4, Associated with Tourette's Disorder. *Mov. Disord.*, **2013**. https://doi.org/10.1002/mds.25460.

[43] Hildonen, M.; Levy, A. M.; Dahl, C.; Bjerregaard, V. A.; Møller, L. B.; Guldberg, P.; Debes, N. M.; Tümer, Z. Elevated Expression of SLC6A4 Encoding the Serotonin Transporter (SERT) in Gilles de La Tourette Syndrome. *Genes (Basel).*, **2021**. https://doi.org/10.3390/genes12010086.

[44] Cao, X.; Zhang, Y.; Abdulkadir, M.; Deng, L.; Fernandez, T. V; Julie, B. G.; Pieter, H.; Robert, J. H.; Justin, A. K.; Kuperman, S.; et al. Whole-Exome Sequencing Identifies Genes Associated with Tourette's Disorder in Multiplex Families. **2021**. https://doi.org/10.1038/s41380-021-01094-1.

[45] Cavallini, M. C.; Di Bella, D.; Catalano, M.; Bellodi, L. An Association Study between 5-HTTLPR Polymorphism, COMT Polymorphism, and Tourette's Syndrome. *Psychiatry Res.*, **2000**. https://doi.org/10.1016/S0165-1781(00)00220-1.

[46] Dehning, S.; Müller, N.; Matz, J.; Bender, A.; Kerle, I.; Benninghoff, J.; Musil, R.; Spellmann, I.; Bondy, B.; Möller, H. J.; et al. A Genetic Variant of HTR2C May Play a Role in the Manifestation of Tourette Syndrome. *Psychiatr. Genet.*, **2010**. https://doi.org/10.1097/YPG.0b013e32833511ce.

[47] Liu, S. G.; Zhang, X. H.; Yin, Y. Y.; Wang, M. J.; Che, F. Y.; Ma, X. An Association Analysis between 5-HTTLPR Polymorphism and Obsessive-Compulsive Disorder, Tourette Syndrome in a Chinese Han Population. *CNS Neurosci. Ther.*, **2011**. https://doi.org/10.1111/j.1755-5949.2011.00274.x.

[48] Comings, D. E.; Muhleman, D.; Dietz, G.; Dino, M.; Legro, R.; Gade, R. Association between Tourette's Syndrome and Homozygosity at the Dopamine D3 Receptor Gene. *Lancet*, **1993**. https://doi.org/10.1016/0140-6736(93)93123-I.

[49] He, F.; Zheng, Y.; Huang, H. H.; Cheng, Y. H.; Wang, C. Y. Association between Tourette Syndrome and the Dopamine D3 Receptor Gene Rs6280. *Chin. Med. J. (Engl).*, **2015**. https://doi.org/10.4103/0366-6999.151665.

[50] Devor, E. J.; Dill-Devor, R. M.; Magee, H. J. The Bal I and Msp I Polymorphisms in the Dopamine D3 Receptor Gene Display, Linkage Disequilibrium with Each Other but No Association with Tourette Syndrome. *Psychiatr. Genet.*, **1998**. https://doi.org/10.1097/00041444-199800820-00003.

[51] Sundaram, S. K.; Huq, A. M.; Wilson, B. J.; Chugani, H. T. Tourette Syndrome Is Associated with Recurrent Exonic Copy Number Variants. *Neurology*, **2010**.

https://doi.org/10.1212/WNL.0b013e3181e0f147.

[52] Bertelsen, B.; Stefánsson, H.; Riff Jensen, L.; Melchior, L.; Mol Debes, N.; Groth, C.; Skov, L.; Werge, T.; Karagiannidis, I.; Tarnok, Z.; et al. Association of AADAC Deletion and Gilles de La Tourette Syndrome in a Large European Cohort. *Biol. Psychiatry*, **2016**. https://doi.org/10.1016/j.biopsych.2015.08.027.

[53] Yuan, L.; Zheng, W.; Yang, Z.; Deng, X.; Song, Z.; Deng, H. Association of the AADAC Gene and Tourette Syndrome in a Han Chinese Cohort. *Neurosci. Lett.*, **2018**. https://doi.org/10.1016/j.neulet.2017.12.034.

[54] Vadgama, N.; Pittman, A.; Simpson, M.; Nirmalananthan, N.; Murray, R.; Yoshikawa, T.; De Rijk, P.; Rees, E.; Kirov, G.; Hughes, D.; et al. De Novo Single-Nucleotide and Copy Number Variation in Discordant Monozygotic Twins Reveals Disease-Related Genes. *Eur. J. Hum. Genet.*, **2019**. https://doi.org/10.1038/s41431-019-0376-7.

[55] Pagliaroli, L.; Vereczkei, A.; Padmanabhuni, S. S.; Tarnok, Z.; Farkas, L.; Nagy, P.; Rizzo, R.; Wolanczyk, T.; Szymanska, U.; Kapisyzi, M.; et al. Association of Genetic Variation in the 3'UTR of LHX6, IMMP2L, and AADAC With Tourette Syndrome. *Front. Neurol.*, **2020**. https://doi.org/10.3389/fneur.2020.00803.

[56] Petek, E.; Windpassinger, C.; Vincent, J. B.; Cheung, J.; Boright, A. P.; Scherer, S. W.; Kroisel, P. M.; Wagner, K. Disruption of a Novel Gene (IMMP2L) by a Breakpoint in 7q31 Associated with Tourette Syndrome. *Am. J. Hum. Genet.*, **2001**. https://doi.org/10.1086/319523.

[57] Patel, C.; Cooper-Charles, L.; McMullan, D. J.; Walker, J. M.; Davison, V.; Morton, J. Translocation Breakpoint at 7q31 Associated with Tics: Further Evidence for IMMP2L as a Candidate Gene for Tourette Syndrome. *Eur. J. Hum. Genet.*, **2011**. https://doi.org/10.1038/ejhg.2010.238.

[58] Bertelsen, B.; Melchior, L.; Jensen, L. R.; Groth, C.; Glenthøj, B.; Rizzo, R.; Debes, N. M.; Skov, L.; Brøndum-Nielsen, K.; Paschou, P.; et al. Intragenic Deletions Affecting Two Alternative Transcripts of the IMMP2L Gene in Patients with Tourette Syndrome. *Eur. J. Hum. Genet.*, **2014**. https://doi.org/10.1038/ejhg.2014.24.

[59] Petek, E.; Schwarzbraun, T.; Noor, A.; Patel, M.; Nakabayashi, K.; Choufani, S.; Windpassinger, C.; Stamenkovic, M.; Robertson, M. M.; Aschauer, H. N.; et al. Molecular and Genomic Studies of IMMP2L and Mutation Screening in Autism and Tourette Syndrome. *Mol. Genet. Genomics*, **2007**. https://doi.org/10.1007/s00438-006-0173-1.

[60] Bjerregaard, V. A.; Schönewolf-Greulich, B.; Juel Rasmussen, L.; Desler, C.; Tümer, Z. Mitochondrial Function in Gilles de La Tourette Syndrome Patients With and Without Intragenic IMMP2L Deletions. *Front. Neurol.*, **2020**. https://doi.org/10.3389/fneur.2020.00163.

[61] Chou, I. C.; Tsai, C. H.; Lee, C. C.; Kuo, H. T.; Hsu, Y. A.; Li, C. I.; Tsai, F. J. Association Analysis between Tourette's Syndrome and Dopamine D1 Receptor Gene in Taiwanese Children. *Psychiatr. Genet.*, **2004**. https://doi.org/10.1097/00041444-200412000-00010.

[62] Gelernter, J.; Kennedy, J. L.; Grandy, D. K.; Zhou, Q. Y.; Civelli, O.; Pauls, D. L.; Pakstis, A.; Kurlan, R.; Sunahara, R. K.; Niznik, H. B.; et al. Exclusion of Close Linkage of Tourette's Syndrome to D1 Dopamine Receptor. *Am. J. Psychiatry*, **1993**. https://doi.org/10.1176/ajp.150.3.449.

[63] Thompson, M.; Comings, D. E.; Feder, L.; George, S. R.; O'Dowd, B. F. Mutation Screening of the Dopamine D1 Receptor Gene in Tourette's Syndrome and Alcohol Dependent Patients. *Am. J. Med. Genet. - Neuropsychiatr. Genet.*, **1998**. https://doi.org/10.1002/(SICI)1096-8628(19980508)81:3<241::AID-AJMG7>3.0.CO;2-Z.

[64] Rivière, J. B.; Xiong, L.; Levchenko, A.; St-Onge, J.; Gaspar, C.; Dion, Y.; Lespérance, P.; Tellier, G.; Richer, F.; Chouinard, S.; et al. Association of Intronic

Variants of the BTBD9 Gene with Tourette Syndrome. *Arch. Neurol.*, **2009**. https://doi.org/10.1001/archneurol.2009.213.

[65] Guo, Y.; Su, L.; Zhang, J.; Lei, J.; Deng, X.; Xu, H.; Yang, Z.; Kuang, S.; Tang, J.; Luo, Z.; et al. Analysis of the BTBD9 and HTR2C Variants in Chinese Han Patients with Tourette Syndrome. *Psychiatr. Genet.*, **2012**. https://doi.org/10.1097/YPG.0b013e32835862b1.

[66] Janik, P.; Berdyński, M.; Safranow, K.; Zekanowski, C. The BTBD9 Gene Polymorphisms in Polish Patients with Gilles de La Tourette Syndrome. *Acta Neurobiol. Exp. (Wars).*, **2014**, *74*, 218–226.

[67] Willsey, A. J.; Fernandez, T. V.; Yu, D.; King, R. A.; Dietrich, A.; Xing, J.; Sanders, S. J.; Mandell, J. D.; Huang, A. Y.; Richer, P.; et al. De Novo Coding Variants Are Strongly Associated with Tourette Disorder. *Neuron*, **2017**. https://doi.org/10.1016/j.neuron.2017.04.024.

[68] Wang, S.; Mandell, J. D.; Kumar, Y.; Sun, N.; Morris, M. T.; Arbelaez, J.; Nasello, C.; Dong, S.; Duhn, C.; Zhao, X.; et al. De Novo Sequence and Copy Number Variants Are Strongly Associated with Tourette Disorder and Implicate Cell Polarity in Pathogenesis. *Cell Rep.*, **2018**. https://doi.org/10.1016/j.celrep.2018.08.082.

[69] Zhao, X.; Wang, S.; Hao, J.; Zhu, P.; Zhang, X.; Wu, M. A Whole-Exome Sequencing Study of Tourette Disorder in a Chinese Population. *DNA Cell Biol.*, **2020**. https://doi.org/10.1089/dna.2019.4746.

[70] Ozbay, F.; Wigg, K. G.; Turanli, E. T.; Asherson, P.; Yazgan, Y.; Sandor, P.; Barr, C. L. Analysis of the Dopamine Beta Hydroxylase Gene in Gilles de La Tourette Syndrome. *Am. J. Med. Genet. Part B Neuropsychiatr. Genet.*, **2006**. https://doi.org/10.1002/ajmg.b.30393.

[71] Mössner, R.; Müller-Vahl, K. R.; Döring, N.; Stuhrmann, M. Role of the Novel Tryptophan Hydroxylase-2 Gene in Tourette Syndrome. *Mol. Psychiatry*, **2007**. https://doi.org/10.1038/sj.mp.4002004.

[72] Zheng, P.; Li, E.; Wang, J.; Cui, X.; Wang, L. Involvement of Tryptophan Hydroxylase 2 Gene Polymorphisms in Susceptibility to Tic Disorder in Chinese Han Population. *Behav. Brain Funct.*, **2013**. https://doi.org/10.1186/1744-9081-9-6.

[73] Abdulkadir, M.; Londono, D.; Gordon, D.; Fernandez, T. V.; Brown, L. W.; Cheon, K. A.; Coffey, B. J.; Elzerman, L.; Fremer, C.; Fründt, O.; et al. Investigation of Previously Implicated Genetic Variants in Chronic Tic Disorders: A Transmission Disequilibrium Test Approach. *Eur. Arch. Psychiatry Clin. Neurosci.*, **2018**. https://doi.org/10.1007/s00406-017-0808-8.

[74] Nag, A.; Bochukova, E. G.; Kremeyer, B.; Campbell, D. D.; Muller, H.; Valencia-Duarte, A. V.; Cardona, J.; Rivas, I. C.; Mesa, S. C.; Cuartas, M.; et al. CNV Analysis in Tourette Syndrome Implicates Large Genomic Rearrangements in COL8A1 and NRXN1. *PLoS One*, **2013**. https://doi.org/10.1371/journal.pone.0059061.

[75] Huang, A. Y.; Yu, D.; Davis, L. K.; Sul, J. H.; Tsetsos, F.; Ramensky, V.; Zelaya, I.; Ramos, E. M.; Osiecki, L.; Chen, J. A.; et al. Rare Copy Number Variants in NRXN1 and CNTN6 Increase Risk for Tourette Syndrome. *Neuron*, **2017**. https://doi.org/10.1016/j.neuron.2017.06.010.

[76] Carias, K. V.; Wevrick, R. Clinical and Genetic Analysis of Children with a Dual Diagnosis of Tourette Syndrome and Autism Spectrum Disorder. *J. Psychiatr. Res.*, **2019**. https://doi.org/10.1016/j.jpsychires.2019.01.023.

[77] Sundaram, S. K.; Huq, A. M.; Sun, Z.; Yu, W.; Bennett, L.; Wilson, B. J.; Behen, M. E.; Chugani, H. T. Exome Sequencing of a Pedigree with Tourette Syndrome or Chronic Tic Disorder. *Ann. Neurol.*, **2011**. https://doi.org/10.1002/ana.22398.

[78] Guo, Y.; Deng, X.; Zhang, J.; Su, L.; Xu, H.; Luo, Z.; Deng, H. Analysis of the MRPL3, DNAJC13 and OFCC1 Variants in Chinese Han Patients with TS-CTD. *Neurosci. Lett.*, **2012**. https://doi.org/10.1016/j.neulet.2012.03.097.

[79] Laurin, N.; Wigg, K. G.; Feng, Y.; Sandor, P.; Barr, C. L. Chromosome 5 and Gilles de La Tourette Syndrome: Linkage in a Large Pedigree and Association Study of Six Candidates in the Region. *Am. J. Med. Genet. Part B Neuropsychiatr. Genet.*, **2009**. https://doi.org/10.1002/ajmg.b.30779.

[80] Lee, P. H.; Anttila, V.; Won, H.; Feng, Y. C. A.; Rosenthal, J.; Zhu, Z.; Tucker-Drob, E. M.; Nivard, M. G.; Grotzinger, A. D.; Posthuma, D.; et al. Genomic Relationships, Novel Loci, and Pleiotropic Mechanisms across Eight Psychiatric Disorders. *Cell*, **2019**. https://doi.org/10.1016/j.cell.2019.11.020.

[81] Tsetsos, F.; Yu, D.; Sul, J. H.; Huang, A. Y.; Illmann, C.; Osiecki, L.; Darrow, S. M.; Hirschtritt, M. E.; Greenberg, E.; Muller-Vahl, K. R.; et al. Synaptic Processes and Immune-Related Pathways Implicated in Tourette Syndrome. *Transl. Psychiatry*, **2021**. https://doi.org/10.1038/s41398-020-01082-z.

[82] Erdmann, J.; Shimron-Abarbanell, D.; Cichon, S.; Albus, M.; Maier, W.; Lichtermann, D.; Minges, J.; Reuner, U.; Franzek, E.; Ertl, M. A.; et al. Systematic Screening for Mutations in the Promoter and the Coding Region of the 5-HT(1A) Gene. *Am. J. Med. Genet. - Neuropsychiatr. Genet.*, **1995**. https://doi.org/10.1002/ajmg.1320600509.

[83] Lam, S.; Shen, Y.; Nguyen, T.; Messier, T. L.; Brann, M.; Comings, D.; George, S. R.; O'Dowd, B. F. A Serotonin Receptor Gene (5HT1A) Variant Found in a Tourette's Syndrome Patient. *Biochem. Biophys. Res. Commun.*, **1996**. https://doi.org/10.1006/bbrc.1996.0322.

[84] Szejko, N.; Fichna, J. P.; Safranow, K.; Dziuba, T.; Żekanowski, C.; Janik, P. Association of a Variant of CNR1 Gene Encoding Cannabinoid Receptor 1 With Gilles de La Tourette Syndrome. *Front. Genet.*, **2020**. https://doi.org/10.3389/fgene.2020.00125.

[85] Gadzicki, D.; Müller-Vahl, K. R.; Heller, D.; Ossege, S.; Nöthen, M. M.; Hebebrand, J.; Stuhrmann, M. Tourette Syndrome Is Not Caused by Mutations in the Central Cannabinoid Receptor (CNR1) Gene. *Am. J. Med. Genet. - Neuropsychiatr. Genet.*, **2004**. https://doi.org/10.1002/ajmg.b.20159.

[86] Verkerk, A. J. M. H.; Mathews, C. A.; Joosse, M.; Eussen, B. H. J.; Heutink, P.; Oostra, B. A.; Tourette Syndrome Association International Consortium for Genetics, T. CNTNAP2 Is Disrupted in a Family with Gilles de La Tourette Syndrome and Obsessive Compulsive Disorder. *Genomics*, **2003**. https://doi.org/10.1016/s0888-7543(03)00097-1.

[87] Belloso, J. M.; Bache, I.; Guitart, M.; Caballin, M. R.; Halgren, C.; Kirchhoff, M.; Ropers, H. H.; Tommerup, N.; Tümer, Z. Disruption of the CNTNAP2 Gene in a t(7;15) Translocation Family without Symptoms of Gilles de La Tourette Syndrome. *Eur. J. Hum. Genet.*, **2007**. https://doi.org/10.1038/sj.ejhg.5201824.

[88] Gade, R.; Muhleman, D.; Blake, H.; MacMurray, J.; Johnson, P.; Verde, R.; Saucier, G.; Comings, D. E. Correlation of Length of VNTR Alleles at the X-Linked MAOA Gene and Phenotypic Effect in Tourette Syndrome and Drug Abuse. *Mol. Psychiatry*, **1998**. https://doi.org/10.1038/sj.mp.4000326.

[89] Bottini, N.; MacMurray, J.; Rostamkani, M.; McGue, M.; Iacono, W. G.; Comings, D. E. Association between the Low Molecular Weight Cytosolic Acid Phosphatase Gene ACP1*A and Comorbid Features of Tourette Syndrome. *Neurosci. Lett.*, **2002**. https://doi.org/10.1016/S0304-3940(02)00750-4.

[90] Yang, Z.; Wu, H.; Lee, P. H.; Tsetsos, F.; Davis, L. K.; Yu, D.; Lee, S. H.; Dalsgaard, S.; Haavik, J.; Barta, C.; et al. Investigating Shared Genetic Basis Across Tourette Syndrome and Comorbid Neurodevelopmental Disorders Along the Impulsivity-Compulsivity Spectrum. *Biol. Psychiatry*, **2021**. https://doi.org/10.1016/j.biopsych.2020.12.028.

[91] Sun, N.; Nasello, C.; Deng, L.; Wang, N.; Zhang, Y.; Xu, Z.; Song, Z.; Kwan, K.; King, R. A.; Pang, Z. P.; et al. The PNKD Gene Is Associated with Tourette Disorder

or Tic Disorder in a Multiplex Family. *Mol. Psychiatry*, **2018**. https://doi.org/10.1038/mp.2017.179.

[92] Adamczyk, A.; Gause, C. D.; Sattler, R.; Vidensky, S.; Rothstein, J. D.; Singer, H.; Wang, T. Genetic and Functional Studies of a Missense Variant in a Glutamate Transporter, SLC1A3, in Tourette Syndrome. *Psychiatr. Genet.*, **2011**. https://doi.org/10.1097/YPG.0b013e328341a307.

[93] Paschou, P.; Stylianopoulou, E.; Karagiannidis, I.; Rizzo, R.; Tarnok, Z.; Wolanczyk, T.; Hebebrand, J.; Nöthen, M. M.; Lehmkuhl, G.; Farkas, L.; et al. Evaluation of the LIM Homeobox Genes LHX6 and LHX8 as Candidates for Tourette Syndrome. *Genes, Brain Behav.*, **2012**. https://doi.org/10.1111/j.1601-183X.2012.00778.x.

[94] Xu, C.; Ozbay, F.; Wigg, K.; Shulman, R.; Tahir, E.; Yazgan, Y.; Sandor, P.; Barr, C. L. Evaluation of the Genes for the Adrenergic Receptors Alpha2A and Alpha1C and Gilles de La Tourette Syndrome. *Am J Med Genet*, **2003**. https://doi.org/10.1002/ajmg.b.20001.

[95] Chou, I. C.; Tsai, C. H.; Wan, L.; Hsu, Y. A.; Tsai, F. J. Association Study between Tourette's Syndrome and Polymorphisms of Noradrenergic Genes (ADRA2A, ADRA2C). *Psychiatr. Genet.*, **2007**. https://doi.org/10.1097/YPG.0b013e3281ac2358.

[96] Eriguchi, Y.; Kuwabara, H.; Inai, A.; Kawakubo, Y.; Nishimura, F.; Kakiuchi, C.; Tochigi, M.; Ohashi, J.; Aoki, N.; Kato, K.; et al. Identification of Candidate Genes Involved in the Etiology of Sporadic Tourette Syndrome by Exome Sequencing. *Am. J. Med. Genet. Part B Neuropsychiatr. Genet.*, **2017**. https://doi.org/10.1002/ajmg.b.32559.

[97] Miranda, D. M.; Wigg, K.; Feng, Y.; Sandor, P.; Barr, C. L. Association Study between Gilles de La Tourette Syndrome and Two Genes in the Robo-Slit Pathway Located in the Chromosome 11q24 Linked/Associated Region. *Am. J. Med. Genet. Part B Neuropsychiatr. Genet.*, **2008**. https://doi.org/10.1002/ajmg.b.30580.

[98] Yu, D.; Sul, J. H.; Tsetsos, F.; Nawaz, M. S.; Huang, A. Y.; Zelaya, I.; Illmann, C.; Osiecki, L.; Darrow, S. M.; Hirschtritt, M. E.; et al. Interrogating the Genetic Determinants of Tourette's Syndrome and Other Tic Disorders through Genome-Wide Association Studies. *Am. J. Psychiatry*, **2019**. https://doi.org/10.1176/appi.ajp.2018.18070857.

[99] Melchior, L.; Bertelsen, B.; Debes, N. M.; Groth, C.; Skov, L.; Mikkelsen, J. D.; Brøndum-Nielsen, K.; Tümer, Z. Microduplication of 15q13.3 and Xq21.31 in a Family with Tourette Syndrome and Comorbidities. *Am. J. Med. Genet. Part B Neuropsychiatr. Genet.*, **2013**. https://doi.org/10.1002/ajmg.b.32186.

[100] Bertelsen, B.; Melchior, L.; Groth, C.; Mol Debes, N.; Skov, L.; Holst, K. K.; Fagerlund, B.; Mikkelsen, J. D.; Tümer, Z. Association of the CHRNA7 Promoter Variant -86T with Tourette Syndrome and Comorbid Obsessive-Compulsive Disorder. *Psychiatry Res.*, **2014**. https://doi.org/10.1016/j.psychres.2014.06.032.

[101] Rippel, C. A.; Kobets, A. J.; Yoon, D. Y.; Williams, P. N.; Shugart, Y. Y.; Bridges, D. D.; Vandenbergh, D. J.; Singer, H. S. Norepinephrine Transporter Polymorphisms in Tourette Syndrome with and without Attention Deficit Hyperactivity Disorder: No Evidence for Significant Association. *Psychiatr. Genet.*, **2006**. https://doi.org/10.1097/01.ypg.0000218622.96127.71.

[102] Crane, J.; Fagerness, J.; Osiecki, L.; Gunnell, B.; Stewart, S. E.; Pauls, D. L.; Scharf, J. M.; Cath, D.; Heutink, P.; Grados, M.; et al. Family-Based Genetic Association Study of DLGAP3 in Tourette Syndrome. *Am. J. Med. Genet. Part B Neuropsychiatr. Genet.*, **2011**. https://doi.org/10.1002/ajmg.b.31134.

[103] Liu, S.; Tian, M.; He, F.; Li, J.; Xie, H.; Liu, W.; Zhang, Y.; Zhang, R.; Yi, M.; Che, F.; et al. Mutations in ASH1L Confer Susceptibility to Tourette Syndrome. *Mol. Psychiatry*, **2020**. https://doi.org/10.1038/s41380-019-0560-8.

[104] Janik, P.; Berdyński, M.; Safranow, K.; Zekanowski, C.; Blum, D. Association of ADORA1 Rs2228079 and ADORA2A Rs5751876 Polymorphisms with Gilles de La Tourette Syndrome in the Polish Population. *PLoS One*, **2015**. https://doi.org/10.1371/journal.pone.0136754.

[105] Liu, W.; Qiu, S.; Gao, C.; Wang, G.; Liu, S.; Guan, H. Lack of Association between SLC5A7 Polymorphisms and Tourette Syndrome in a Chinese Han Population. *Neurosci. Lett.*, **2017**. https://doi.org/10.1016/j.neulet.2017.08.041.

[106] Chou, I. C.; Lin, H. C.; Wang, C. H.; Lin, W. De; Lee, C. C.; Tsai, C. H.; Tsai, F. J. Polymorphisms of Interleukin 1 Gene IL1RN Are Associated With Tourette Syndrome. *Pediatr. Neurol.*, **2010**. https://doi.org/10.1016/j.pediatrneurol.2010.01.006.

[107] Yuan, A.; Wang, Z.; Xu, W.; Ding, Q.; Zhao, Y.; Han, J.; Sun, J. A Rare Novel CLCN2 Variation and Risk of Gilles de La Tourette Syndrome: Whole-Exome Sequencing in a Multiplex Family and a Follow-Up Study in a Chinese Population. *Front. Psychiatry*, **2020**. https://doi.org/10.3389/fpsyt.2020.543911.

[108] Huang, Y.; Li, T.; Wang, Y.; Ansar, J.; Lanting, G.; Liu, X.; Zhao, J. H.; Hu, X.; Sham, P. C.; Collier, D. Linkage Disequilibrium Analysis of Polymorphisms in the Gene for Myelin Oligodendrocyte Glycoprotein in Tourette's Syndrome Patients from a Chinese Sample. *Am. J. Med. Genet. - Neuropsychiatr. Genet.*, **2004**. https://doi.org/10.1002/ajmg.b.20079.

[109] Prontera, P.; Napolioni, V.; Ottaviani, V.; Rogaia, D.; Fusco, C.; Augello, B.; Serino, D.; Parisi, V.; Bernardini, L.; Merla, G.; et al. DPP6 Gene Disruption in a Family with Gilles de La Tourette Syndrome. *Neurogenetics*, **2014**. https://doi.org/10.1007/s10048-014-0418-9.

[110] Matsumoto, N.; David, D. E.; Johnson, E. W.; Konecki, D.; Burmester, J. K.; Ledbetter, D. H.; Weber, J. L. Breakpoint Sequences of an 1;8 Translocation in a Family with Gilles de La Tourette Syndrome. *Eur. J. Hum. Genet.*, **2000**. https://doi.org/10.1038/sj.ejhg.5200549.

[111] Scharf, J. M.; Yu, D.; Mathews, C. A.; Neale, B. M.; Stewart, S. E.; Fagerness, J. A.; Evans, P.; Gamazon, E.; Edlund, C. K.; Service, S. K.; et al. Genome-Wide Association Study of Tourette's Syndrome. *Mol. Psychiatry*, **2013**. https://doi.org/10.1038/mp.2012.69.

[112] Bertelsen, B.; Melchior, L.; Jensen, L. R.; Groth, C.; Nazaryan, L.; Debes, N. M.; Skov, L.; Xie, G.; Sun, W.; Brøndum-Nielsen, K.; et al. A t(3;9)(Q25.1;Q34.3) Translocation Leading to OLFM1 Fusion Transcripts in Gilles de La Tourette Syndrome, OCD and ADHD. *Psychiatry Res.*, **2015**. https://doi.org/10.1016/j.psychres.2014.12.028.

[113] Kindler, J.; Schosser, A.; Stamenkovic, M.; Schloegelhofer, M.; Leisch, F.; Hornik, K.; Aschauer, H.; Gasche, C. Tourette's Syndrome Is Not Associated with Interleukin-10 Receptor 1 Variants on Chromosome 11q23.3. *Psychiatry Res.*, **2008**. https://doi.org/10.1016/j.psychres.2007.07.021.

[114] Niesler, B.; Frank, B.; Hebebrand, J.; Rappold, G. Serotonin Receptor Genes HTR3A and HTR3B Are Not Involved in Gilles de La Tourette Syndrome. *Psychiatr. Genet.*, **2005**. https://doi.org/10.1097/00041444-200512000-00015.

[115] Liu, W.; Zhang, X.; Deng, Z.; Li, G.; Zhang, R.; Yang, Z.; Che, F.; Liu, S.; Li, H. The Role of SLITRK6 in the Pathogenesis of Tourette Syndrome: From the Conclusion of a Family-Based Study in the Chinese Han Population. *J. Gene Med.*, **2020**. https://doi.org/10.1002/jgm.3173.

[116] Zhang, K.; Feng, Y.; Wigg, K. G.; Sandor, P.; Barr, C. L. Association Study of the SLITRK5 Gene and Tourette Syndrome. *Psychiatr. Genet.*, **2015**. https://doi.org/10.1097/YPG.0000000000000067.

[117] Liu, W.; Guo, Y.; Liu, X.; Zhang, R.; Dong, J.; Deng, H.; He, F.; Che, F.; Liu, S.; Yi, M. Family-Based Analysis Combined with Case–Controls Study Implicate Roles of PCNT in Tourette Syndrome. *Neuropsychiatr. Dis. Treat.*, **2020**. https://doi.org/10.2147/NDT.S229420.

[118] Lawson-Yuen, A.; Saldivar, J. S.; Sommer, S.; Picker, J. Familial Deletion within NLGN4 Associated with Autism and Tourette Syndrome. *Eur. J. Hum. Genet.*, **2008**. https://doi.org/10.1038/sj.ejhg.5202006.

[119] Barr, C. L.; Wigg, K. G.; Pakstis, A. J.; Kurlan, R.; Pauls, D.; Kidd, K. K.; Tsui, L. C.; Sandor, P. Genome Scan for Linkage to Gilles de La Tourette Syndrome. *Am. J. Med. Genet. - Neuropsychiatr. Genet.*, **1999**. https://doi.org/10.1002/(SICI)1096-8628(19990820)88:4<437::AID-AJMG24>3.0.CO;2-E.

[120] Rivière, J. B.; St-Onge, J.; Gaspar, C.; Diab, S.; Dion, Y.; Lespérance, P.; Tellier, G.; Richer, F.; Chouinard, S.; Dubé, M. P.; et al. Genome-Wide TDT Analysis in French-Canadian Families with Tourette Syndrome. *Can. J. Neurol. Sci.*, **2010**. https://doi.org/10.1017/S0317167100009744.

[121] Yu, D.; Mathews, C. A.; Scharf, J. M.; Neale, B. M.; Davis, L. K.; Gamazon, E. R.; Derks, E. M.; Evans, P.; Edlund, C. K.; Crane, J.; et al. Cross-Disorder Genome-Wide Analyses Suggest a Complex Genetic Relationship between Tourette's Syndrome and OCD. *Am. J. Psychiatry*, **2015**. https://doi.org/10.1176/appi.ajp.2014.13101306.

[122] Zilhão, N. R.; Padmanabhuni, S. S.; Pagliaroli, L.; Barta, C.; Smit, D. J. A.; Cath, D.; Nivard, M. G.; Baselmans, B. M. L.; Van Dongen, J.; Paschou, P.; et al. Epigenome-Wide Association Study of Tic Disorders. *Twin Res. Hum. Genet.*, **2015**. https://doi.org/10.1017/thg.2015.72.

[123] Rizzo, R.; Ragusa, M.; Barbagallo, C.; Sammito, M.; Gulisano, M.; Calì, P. V.; Pappalardo, C.; Barchitta, M.; Granata, M.; Condorelli, A. G.; et al. Circulating MiRNAs Profiles in Tourette Syndrome: Molecular Data and Clinical Implications. *Mol. Brain*, **2015**. https://doi.org/10.1186/s13041-015-0133-y.

[124] Knight, S.; Coon, H.; Johnson, M.; Leppert, M. F.; Camp, N. J.; McMahon, W. M.; Cath, D.; Heutink, P.; Grados, M.; Singer, H. S.; et al. Linkage Analysis of Tourette Syndrome in a Large Utah Pedigree. *Am. J. Med. Genet. Part B Neuropsychiatr. Genet.*, **2010**. https://doi.org/10.1002/ajmg.b.31035.

[125] Pauls, D.; Cath, D.; Heutink, P.; Grados, M.; Singer, H. S.; Walkup, J. T.; Illmann, C.; Scharf, J. M.; Santangelo, S.; Stewart, S. E.; et al. Genome Scan for Tourette Disorder in Affected-Sibling-Pair and Multigenerational Families. *Am. J. Hum. Genet.*, **2007**. https://doi.org/10.1086/511052.

[126] Simonic, I.; Nyholt, D. R.; Gericke, G. S.; Gordon, D.; Matsumoto, N.; Ledbetter, D. H.; Ott, J.; Weber, J. L. Rapid Publication: Further Evidence for Linkage of Gilles de La Tourette Syndrome (GTS) Susceptibility Loci on Chromosomes 2p11, 8q22, and 11q23-24 in South African Afrikaners. *Am. J. Med. Genet. - Neuropsychiatr. Genet.*, **2001**. https://doi.org/10.1002/ajmg.1192.

[127] Simonic, I.; Gericke, G. S.; Ott, J.; Weber, J. L. Identification of Genetic Markers Associated with Gilles de La Tourette Syndrome in an Afrikaner Population. *Am. J. Hum. Genet.*, **1998**. https://doi.org/10.1086/302002.

[128] Brett, P.; Curtis, D.; Gourdie, A.; Schnieden, V.; Jackson, G.; Holmes, D.; Robertson, M.; Gurling, H. Possible Linkage of Tourette Syndrome to Markers on Short Arm of Chromosome 3 (C3p21-14). *Lancet*, **1990**. https://doi.org/10.1016/0140-6736(90)92555-V.

[129] Verkerk, A. J. M. H.; Cath, D. C.; Van Der Linde, H. C.; Both, J.; Heutink, P.; Breedveld, G.; Aulchenko, Y. S.; Oostra, B. A. Genetic and Clinical Analysis of a Large Dutch Gilles de La Tourette Family. *Mol. Psychiatry*, **2006**. https://doi.org/10.1038/sj.mp.4001877.

[130] Pauls, D. A Complete Genome Screen in Sib Pairs Affected by Gilles de La Tourette Syndrome. *Am. J. Hum. Genet.*, **1999**. https://doi.org/10.1086/302613.

[131] Zhang, H.; Leckman, J. F.; Pauls, D. L.; Tsai, C. P.; Kidd, K. K.; Rosario Campos, M. Genomewide Scan of Hoarding in Sib Pairs in Which Both Sibs Have Gilles de La Tourette Syndrome. *Am. J. Hum. Genet.*, **2002**. https://doi.org/10.1086/339520.

[132] Curtis, D.; Brett, P.; Dearlove, A. M.; McQuillin, A.; Kalsi, G.; Robertson, M. M.; Gurling, H. M. D. Genome Scan of Tourette Syndrome in a Single Large Pedigree Shows Some Support for Linkage to Regions of Chromosomes 5, 10 and 13. *Psychiatr. Genet.*, **2004**. https://doi.org/10.1097/01.ypg.0000107927.32051.f5.

[133] Díaz-Anzaldúa, A.; Joober, R.; Rivière, J. B.; Dion, Y.; Lespérance, P.; Chouinard, S.; Richer, F.; Rouleau, G. A. Association between 7q31 Markers and Tourette Syndrome. *Am. J. Med. Genet.*, **2004**. https://doi.org/10.1002/ajmg.a.20631.

[134] Díaz-Anzaldúa, A.; Rivière, J. B.; Dubé, M. P.; Joober, R.; Saint-Onge, J.; Dion, Y.; Lespérance, P.; Richer, F.; Chouinard, S.; Rouleau, G. A.; et al. Chromosome H-Q24 Region in Tourette Syndrome: Association and Linkage Disequilibrium Study in the French Canadian Population. *Am. J. Med. Genet.*, **2005**, *138 A* (3), 225–228. https://doi.org/10.1002/ajmg.a.30928.

[135] Mérette, C.; Brassard, A.; Potvin, A.; Bouvier, H.; Rousseau, F.; Édmond, C.; Bissonnette, L.; Roy, M. A.; Maziade, M.; Ott, J.; et al. Significant Linkage for Tourette Syndrome in a Large French Canadian Family. *Am. J. Hum. Genet.*, **2000**. https://doi.org/10.1086/303093.

[136] Paschou, P.; Yu, D.; Gerber, G.; Evans, P.; Tsetsos, F.; Davis, L. K.; Karagiannidis, I.; Chaponis, J.; Gamazon, E.; Mueller-Vahl, K.; et al. Genetic Association Signal near NTN4 in Tourette Syndrome. *Ann. Neurol.*, **2014**. https://doi.org/10.1002/ana.24215.

[137] Padmanabhuni, S. S.; Houssari, R.; Esserlind, A. L.; Olesen, J.; Werge, T. M.; Hansen, T. F.; Bertelsen, B.; Tsetsos, F.; Paschou, P.; Tümer, Z. Investigation of SNP Rs2060546 Immediately Upstream to NTN4 in a Danish Gilles de La Tourette Syndrome Cohort. *Front. Neurosci.*, **2016**. https://doi.org/10.3389/fnins.2016.00531.

[138] Breedveld, G. J.; Fabbrini, G.; Oostra, B. A.; Berardelli, A.; Bonifati, V. Tourette Disorder Spectrum Maps to Chromosome 14q31.1 in an Italian Kindred. *Neurogenetics*, **2010**. https://doi.org/10.1007/s10048-010-0244-7.

[139] McGrath, L. M.; Yu, D.; Marshall, C.; Davis, L. K.; Thiruvahindrapuram, B.; Li, B.; Cappi, C.; Gerber, G.; Wolf, A.; Schroeder, F. A.; et al. Copy Number Variation in Obsessive-Compulsive Disorder and Tourette Syndrome: A Cross-Disorder Study. *J. Am. Acad. Child Adolesc. Psychiatry*, **2014**. https://doi.org/10.1016/j.jaac.2014.04.022.

[140] Shelley, B. P.; Robertson, M. M.; Turk, J. An Individual with Gilles de La Tourette Syndrome and Smith-Magenis Microdeletion Syndrome: Is Chromosome 17p11.2 a Candidate Region for Tourette Syndrome Putative Susceptibility Genes? *J. Intellect. Disabil. Res.*, **2007**. https://doi.org/10.1111/j.1365-2788.2006.00943.x.

[141] Paschou, P.; Feng, Y.; Pakstis, A. J.; Speed, W. C.; DeMille, M. M.; Kidd, J. R.; Jaghori, B.; Kurlan, R.; Pauls, D. L.; Sandor, P.; et al. Indications of Linkage and Association of Gilles de La Tourette Syndrome in Two Independent Family Samples: 17q25 Is a Putative Susceptibility Region. *Am. J. Hum. Genet.*, **2004**. https://doi.org/10.1086/424389.

[142] Robertson, M. M.; Shelley, B. P.; Dalwai, S.; Brewer, C.; Critchley, H. D. A Patient with Both Gilles de La Tourette's Syndrome and Chromosome 22q11 Deletion Syndrome: Clue to the Genetics of Gilles de La Tourette's Syndrome? *J. Psychosom. Res.*, **2006**. https://doi.org/10.1016/j.jpsychores.2006.06.011.

[143] Maccarini, S.; Cipani, A.; Bertini, V.; Skripac, J.; Salvi, A.; Borsani, G.; Marchina, E. Inherited Duplication of the Pseudoautosomal Region Xq28 in a Subject with Gilles de La Tourette Syndrome and Intellectual Disability: A Case Report. *Mol. Cytogenet.*, **2020**. https://doi.org/10.1186/s13039-020-00493-3.

[144] Kroisel, P. M.; Petek, E.; Emberger, W.; Windpassinger, C.; Wladika, W.; Wagner, K. Candidate Region for Gilles de La Tourette Syndrome at 7q31. *Am J Med*

Genet, **2001**.

[145] Crawford, F. C.; Ait-Ghezala, G.; Morris, M.; Sutcliffe, M. J.; Hauser, R. A.; Silver, A. A.; Mullan, M. J. Translocation Breakpoint in Two Unrelated Tourette Syndrome Cases, within a Region Previously Linked to the Disorder. *Hum. Genet.*, **2003**. https://doi.org/10.1007/s00439-003-0942-4.

[146] State, M. W.; Greally, J. M.; Cuker, A.; Bowers, P. N.; Henegariu, O.; Morgan, T. M.; Gunel, M.; DiLuna, M.; King, R. A.; Nelson, C.; et al. Epigenetic Abnormalities Associated with a Chromosome 18(Q21-Q22) Inversion and a Gilles de La Tourette Syndrome Phenotype. *Proc. Natl. Acad. Sci. U. S. A.*, **2003**. https://doi.org/10.1073/pnas.0730775100.

[147] Jankovic, J.; Deng, H. Candidate Locus for Chorea and Tic Disorders at 15q? *Pediatr. Neurol.*, **2007**. https://doi.org/10.1016/j.pediatrneurol.2007.02.015.

[148] Dehning, S.; Riedel, M.; Müller, N. Father-to-Son Transmission of 6;17 Translocation in Tourette's Syndrome. *Am. J. Psychiatry*, **2008**. https://doi.org/10.1176/appi.ajp.2008.07111828.

[149] Hooper, S. D.; Johansson, A. C. V.; Tellgren-Roth, C.; Stattin, E. L.; Dahl, N.; Cavelier, L.; Feuk, L. Genome-Wide Sequencing for the Identification of Rearrangements Associated with Tourette Syndrome and Obsessive-Compulsive Disorder. *BMC Med. Genet.*, **2012**. https://doi.org/10.1186/1471-2350-13-123.

[150] Bertelsen, B.; Debes, N. M.; Hjermind, L. E.; Skov, L.; Brøndum-Nielsen, K.; Tümer, Z. Chromosomal Rearrangements in Tourette Syndrome: Implications for Identification of Candidate Susceptibility Genes and Review of the Literature. *Neurogenetics*, **2013**. https://doi.org/10.1007/s10048-013-0372-y.
